# Contrasting molecular and pharmacodynamic signatures of response and resistance to sequential bortezomib and temozolomide in recurrent *IDH*-wildtype glioblastoma and grade 4 *IDH*-mutant astrocytoma: an exploratory interim analysis of NCT03643549 phase IB/II trial

**DOI:** 10.1186/s40364-026-01023-w

**Published:** 2026-10-27

**Authors:** Dorota Goplen, Mohummad Aminur Rahman, Jorunn Brekke, Andreas Waha, Leif Oltedal, Judit Haasz, Surendra Kumar, Even Birkeland, Frode Selheim, Stein Atle Lie, Martha Chekenya

**Affiliations:** 1 Department of Oncology, Haukeland University Hospital, Bergen, Norway; 2 Department of Clinical Medicine, University of Bergen, Bergen, Norway; 3 Department of Biomedicine, University of Bergen, Bergen, Norway; 4 Department of Neuropathology, University of Bonn, Bonn, Germany; 5 Mohn Medical Imaging and Visualization Centre, Department of Radiology, Haukeland University Hospital, Bergen, Norway; 6 Newfoundland and Labrador Health Services, Department of Research and Innovation, Newfoundland, Canada; 7 Department of Clinical Dentistry, University of Bergen, Bergen, Norway

**Keywords:** Amplified *EGFR*, Activated NFkB, Proteasome inhibitor, *MGMT* methylation, *CDKN2A/RB1*-cell-cycle regulatory axis

## Abstract

**Background:**

The revision of the WHO classification of CNS tumours now distinguishes grade 4 diffuse gliomas by their molecular features, but tailored treatment is yet to be standardised. Glioblastoma (isocitrate dehydrogenase (*IDH*))-wildtype tumours harbouring an unmethylated *O*^6^-methylguanine-DNA methyltransferase “(u*MGMT*)” promoter are inherently resistant to temozolomide (TMZ). However, no standardised treatment recommendations are available for grade 4 astrocytoma *IDH*-mutant. We previously demonstrated that administration of bortezomib (BTZ) 48 h prior to TMZ depletes MGMT protein and sensitizes u*MGMT* glioma cells to TMZ. Based on these findings, a phase IB/II trial was initiated to evaluate the safety and clinical activity of sequential BTZ and TMZ. This interim analysis aimed to determine a futility safeguard, identify molecular and pharmacodynamic determinants of treatment response and failure in the first 15 patients consecutively treated.

**Methods:**

Adult patients with recurrent grade 4 *IDH-*mutant astrocytoma or *IDH-*wildtype glioblastoma, u*MGMT* promoter, measurable disease, Karnofsky Performance Status ≥ 70, and disease progression ≥ 12 weeks after radiotherapy were enrolled. Patients received intravenous BTZ 1.3 mg/m² on days 1, 4, and 7 of each 28-day cycle, followed by oral TMZ 200 mg/m²/day for 5 consecutive days beginning on day 3. Targeted sequencing of glioma-associated genes and longitudinal plasma proteomic profiling using LC-MS/MS were performed before, during, and after treatment.

**Results:**

Fifteen patients (median age 52 years) comprised 10 patients with *IDH*- wildtype glioblastoma and 5 patients with grade 4 *IDH*-mutant astrocytoma. Disease control (partial response and stable disease) was observed in 4/10 (40%) *IDH*- wildtype glioblastoma patients, comprising one PR and three cases of SD. In contrast, all five patients with grade 4 *IDH*-mutant astrocytoma experienced rapid progressive disease. Notably, non-responding *IDH*-wildtype glioblastomas and all *IDH*-mutant tumours harboured alterations affecting the *CDKN2A/RB1*-cell-cycle regulatory axis, including *CDKN2A* and/or *RB1*loss, with one *IDH*-mutant patient additionally harbouring *PIK3CA* gain. By comparison, responding *IDH-*wildtype glioblastomas were characterized by amplified *EGFR*, nuclear NF-κB (p65/RelA) activation, and absence of *RB-*pathway alterations. Longitudinal proteomic analyses demonstrated comparatively attenuated treatment-induced changes in proteins associated with invasion and extracellular matrix remodelling in responders, whereas proteins involved in damage clearance immune phenotype, platelet activation, aggregation, and degranulation were significantly altered during disease control.

**Conclusions:**

Sequential BTZ and TMZ therapy was feasible with manageable hematologic and non-hematologic toxicities and met prespecified interim futility criteria, supporting continued recruitment to the phase II study. Disease control was restricted to a molecularly defined subset of *IDH*- wildtype glioblastoma characterized by *EGFR* amplification and NF-κB activation. In contrast, universal treatment failure among grade 4 *IDH*-mutant astrocytomas was associated with alterations of the *CDKN2A/RB1* cell-cycle checkpoint pathway, suggesting that advanced molecular evolution and cell-cycle deregulation may override the survival advantage typically associated with *IDH*-mutant gliomas and limit the effectiveness of MGMT-targeted sensitization strategies.

**Registry:**

ClinicalTrials.gov trial registration number: NCT03643549, date of registration: 2018-08-21.

**Supplementary Information:**

The online version contains supplementary material available at https://doi.org/10.1186/s40364-026-01023-w.

## Introduction

Diffuse gliomas represent the most common malignant primary brain tumours in adults [1]. The 2021 World Health Organisation classification of tumours of the central nervous system (WHO CNS5) [1] now recognises grade 4 adult-type diffuse gliomas as two biologically distinct disease entities, most notably glioblastoma (GBM) carrying wild type isocitrate dehydrogenase (*IDH1/2* genes, *IDH*-wildtype), and astrocytoma *IDH*-mutant after acquisition of high-risk molecular alterations [1, 2]. Although both entities are aggressive, they differ substantially in molecular pathogenesis, prognosis and mechanisms of therapeutic resistance. *IDH*-wildtype glioblastoma is the most common in adults, characterized by rapid infiltrative growth and marked intratumoral heterogeneity, where median overall survival is 9–12 months [3, 4], with 5-year survival rates below 10% [5, 6]. Although grade 4 *IDH*-mutant astrocytomas exhibit more prolonged clinical course, until recently, the standard-of-care for newly diagnosed grade 4 gliomas has been similar to that of *IDH*-wildtype glioblastoma, consisting of maximal safe surgical resection, followed by daily temozolomide (TMZ) chemotherapy for 6 weeks administered concurrently with fractionated conformal ionising radiation (IR) to total 60 Gy, and thereafter adjuvant TMZ for 5 days every 28 days for upto six cycles [7]. Emerging findings indicate lack of association of *MGMT* promoter methylation or TMZ response with improved overall survival [8, 9]. While exploratory analyses had found no benefit of TMZ administered concurrently with IR in patients harbouring *IDH*-mutant astrocytoma, the randomised CATNON trial demonstrated benefit of 12 cycles adjuvant TMZ in grade 3 *IDH*-mutant astrocytoma, suggesting this prolonged adjuvant TMZ treatment be considered for also for grade 4 *IDH*-mutant astrocytomas [10]. Nevertheless, recurrence remains inevitable and there is no universally accepted standard treatment at progression. The European Society for Medical Oncology (ESMO) guidelines recommend enrolment in clinical trials whenever possible [11].

Prior to implementation of the WHO CNS5 classification system [1], the consensus has been that resistance to TMZ remains the major obstacle to improved outcomes. TMZ exerts its cytotoxic effect primarily through DNA alkylation at the O^6^ position of guanine, a lesion repaired by the DNA repair enzyme O^6^-methylguanine-DNA methyltransferase (*MGMT*). Methylation of the *MGMT* promoter silences expression of the DNA repair enzyme, rendering tumours more susceptible to TMZ chemotherapy and improved outcome. Because 50–60% of GBM patients harbour an unmethylated *MGMT* promoter (u*MGMT*), which is both predictive for poor response to TMZ and prognostic for reduced survival [12, 13], strategies capable of suppressing MGMT expression or function remain clinically important in this subset. The 26S proteasome regulates the signalling, stability, and turnover of multiple proteins involved in DNA repair, cell survival, inflammation, apoptosis, and tumour adaptation in an ATP-dependent manner [14, 15]. Proteasome inhibition has therefore emerged as a rational strategy to perturb resistance networks in glioma. We previously demonstrated that 48-hour pre-treatment with bortezomib (BTZ), a reversible proteasome inhibitor approved for multiple myeloma [16], depletes MGMT mRNA and protein expression, disrupts pro-survival autophagic flux, reduces NF-κB signalling, and sensitizes unmethylated *MGMT* glioblastoma cells to TMZ [15, 17]. Others showed it modulated MAPK, STAT3 signalling, decreased BCL-2 and induced apoptosis [17, 18, 15]. Our subsequent phase IB dose escalation demonstrated that the safety profile of sequential BTZ followed by TMZ was manageable, with platelet nadirs consistently recovering by day 22 of each treatment cycle [19].

Although *MGMT* promoter status is an important determinant of TMZ sensitivity, treatment response in recurrent disease is likely influenced by broader tumour biology. Recurrent gliomas are modulated by prior therapy, intratumoral heterogeneity through clonal selection and evolving molecular alterations. Recent studies have highlighted the prognostic importance of molecular features and propose consideration of additional molecular alterations such as homozygous *CDKN2A/B* deletion and others that affect cell-cycle and growth regulatory pathways to modify prognosis and therapeutic response of grade 4 tumours [1, 2, 8, 10].

This interim analysis of the first 15 patients enrolled in the NCT03643549 phase IB/II trial that was initiated before the current WHO CNS5 classification offers the opportunity to evaluate the safety, preliminary clinical activity, and exploratory molecular and pharmacodynamic correlates of sequential BTZ and TMZ in distinct recurrent grade 4 gliomas with unmethylated *MGMT* promoter, while preserving the original therapeutic rationale. Targeted sequencing and longitudinal plasma proteomic profiling were performed to identify biological features associated with treatment response or progression and to inform further evaluation in the ongoing phase II cohort.

## Results

### Interim patient characteristics

**Fig. 1:**
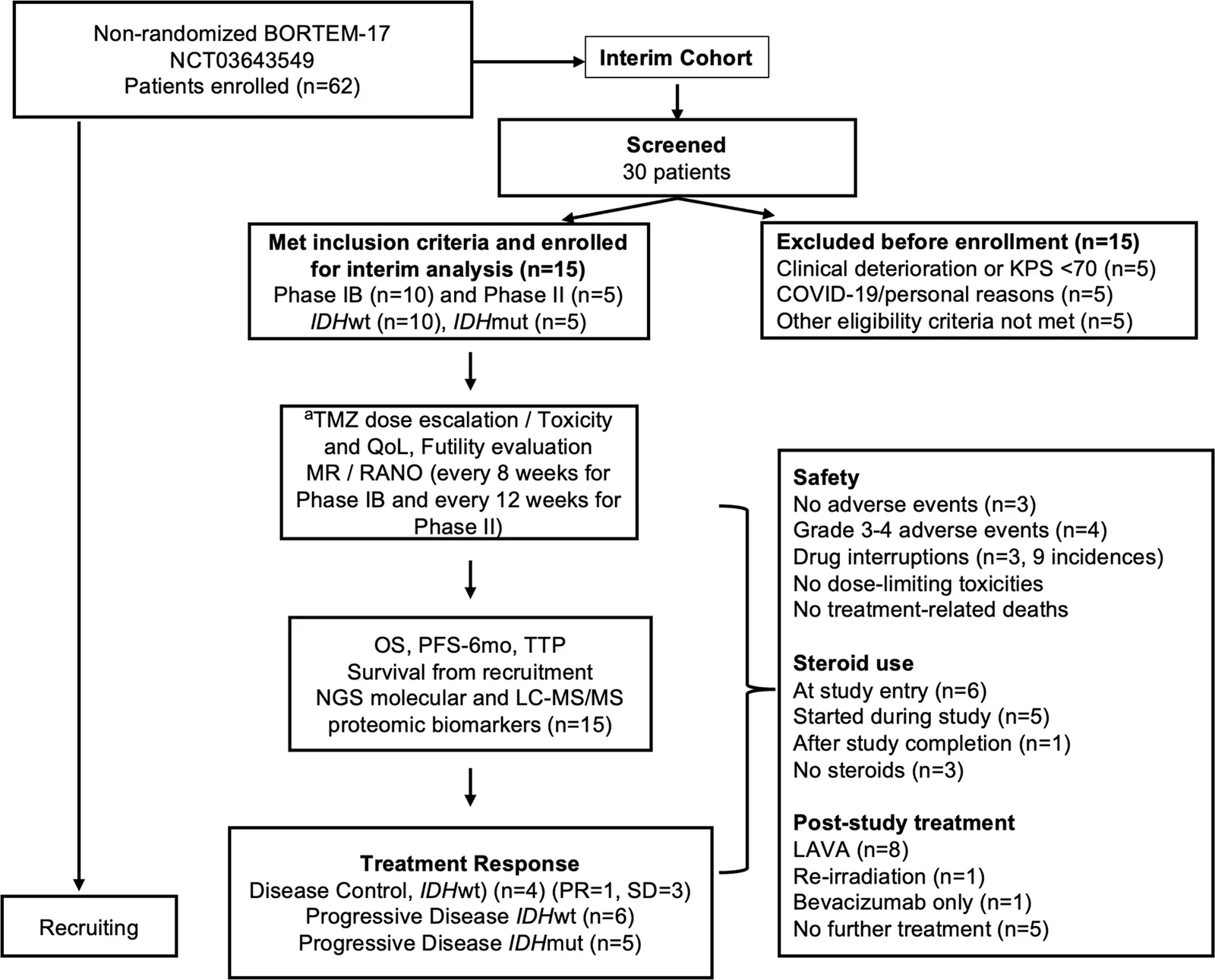
Flowchart of interim patient cohort composition, incident, safety, and treatment response. The interim cohort comprised 15 patients, including 10 with IDH-wildtype GBM and five with grade 4 IDH-mutant astrocytoma. Three patients had nine treatment interruptions, but no dose-limiting toxicities or treatment-related deaths were observed. TMZ= Temozolomide, QoL= Quality of life, MR= Magnetic resonance, RANO= Response assessment in neuro-oncology, IDH= isocitrate dehydrogenase, OS= Overall survival (survival from diagnosis to death), PFS-6mo= Progression free survival at 6 months, TTP= Time to progression. NGS= Next generation sequencing, LC-MS/MS= Liquid chromatography mass spectrometry, KPS= Karnofsky performance status, PCV= Combination of Procarbazine, Lomustine and Vincristine, COVID= Corona virus disease, BTZ= Bortezomib, DLT= Dose limiting toxicity, AE= Adverse events, LAVA= combination of Lomustin 100 mg/m^2^ adjusted to whole 40 mg capsules day 1, Avastin 400 mg (flat dose) day 1 and 15, Vincristine 2 mg flat dose day 1, 6 weeks cycle

The interim cohort comprised the first 15 consecutively enrolled patients in the BORTEM-17 trial, all recruited between September 2018 to June 2021 according to the same inclusion criteria, Fig. 1. These patients were pre-defined for interim analysis to determine whether the study should proceed to full accrual of the *n* = 63 trial cohort. The protocol prespecified that the trial would be terminated if none of these patients derived clinical benefit, defined as disease control rate (DCR) according to RANO criteria for partial response (PR) or stable disease (SD), to avoid exposure to ineffective therapy. We used DCR including SD, rather than the standard Objective Response Rate (ORR; CR + PR) as the primary futility endpoint because the trial enrolled grade 4 glioma patients with recurrent u*MGMT* tumours with particularly dismal prognosis, rapidly progressing after standard of care and deteriorating clinical status. These patients are known historically to gain limited benefit to temozolomide [20] and with paucity of viable therapeutic options. In this context stabilization of both clinical and radiological findings is considered as clinical benefit. As the protocol permitted patients with expected life expectancy of 8 weeks and CR has never been achieved in this patient group [6], so stable disease was considered an acceptable response outcome. Response was assessed after at least two treatment cycles, or earlier if clinically indicated, and treatment was discontinued upon MRI-confirmed disease progression. Therefore, only patients with clinical benefit after having received at least two cycles of treatment were evaluable for response.

The summarised clinical and molecular characteristics of the 15 patients included in the interim analysis are shown in Table 1. The median age of the patients was 52 years (range, 25–62). The patient cohort included 10 males and 5 females. Median KPS was 90 (range, 80–100) and median NANO score was 1 (range, 0–7). Half of the patients had gross total tumour resection, and half underwent subtotal resection. A total of 42 BORTEM cycles were administered; the median number of cycles was 2 (range, 2–6), and the median TMZ dose was 187.5 mg/m^2^.

**Table 1:** Patient's characteristics

ID	Age	Gender	% of MGMT methylation (pyrosequencing) mean ± SEM	Molecular profiles					MedianTMZ dose (mg/m^2^)	Treatment Response	Post Withdrawal Treatment	Survival from Recruitment (months)	Overall Survival from diagnosis (months)
				IDH	EGFR	RB1	CDKN2A	PIK3CA					
P03	53	M	2±0.6	WT	WT	WT	WT	WT	200	SD	Re-irradiated	14.5	33.8
P11	62	F	4±2.1	WT	Gain	WT	WT	WT	175	PR	No treatment	5.4	13.4
P12	49	M	3±0.6	WT	Gain	WT	Loss	WT	150	SD	LAVA	20.9	41.0
P13	55	F	19±4	WT	Gain	WT	Loss	WT	140	SD	No treatment	2.5	23.7
P01	46	M	3±1.1	Mut	WT	WT	Loss	WT	175	PD	LAVA	6.6	27.3
P02	54	M	2±0.6	WT	WT	Loss	Loss	WT	162.5	PD	Bevacizumab only	6.2	24.0
P04	39	F	3±1.7	Mut	WT	Loss	WT	WT	187.5	PD	No treatment	3.8	13.8
P05	34	M	2±0.3	Mut	WT	Loss	WT	WT	187.5	PD	LAVA	23.8	39.9
P06	34	F	3±0.7	WT	WT	Loss	Loss	Gain	150	PD	LAVA	3.2	15.2
P07	25	M	1±0.2	Mut	Gain	WT	Loss	Gain	187.5	PD	LAVA	15.0	32.4
P08	56	M	2±0.4	WT	WT	Loss	WT	WT	200	PD	LAVA	4.2	18.7
P09	57	M	2±0.8	WT	WT	WT	Loss	WT	200	PD	No treatment	2.2	12.8
P10	52	M	4±1.3	WT	WT	WT	Loss	WT	200	PD	No treatment	2.4	11.2
P14	58	M	2±0.3	WT	WT	Loss	Loss	WT	200	PD	LAVA	8.2	25.9
P15	49	F	5±0.9	Mut	WT	Loss	Loss	WT	200	PD	LAVA	14.3	28.7
**Overall Characteristics**													
Age	n	%	Gender	n	%	Surgery		n	%	KPS	n	%	
21-40	4	26.7	Male	10	66.7	GTR		7	46.7	80	7	46.7	
41-60	10	66.7	Female	5	33.3	STR		7	46.7	90	6	40.0	
61-70	1	6.7	-	-	-	Biopsy		1	6.7	100	2	13.3	

M=male, F=female, WT= wild-type, Mut=mutated, GTR=gross total resection, STR=sub-total resection, TMZ=temozolomide, LAVA=combination of Lomustin 100 mg/m^2^ adjusted to whole 40 mg capsules day 1, Avastin 400 mg (flat dose) day 1 and 15, Vincristine 2 mg flat dose day 1, 6 weeks cycle. SD=stable disease, PR=partial response, PD=progressive disease.The first four highlighted patients are the patients achieving disease control (PR+SD)

### Simon’s MinMax-like design and disease control evaluation

Out of the first 15 patients treated, three achieved SD (6 months (P03); 8 months (P12) and 2 months (P13)), and 1 achieved PR (4 months (P11)). Binomial exact confidence intervals for (n= 15) was ORR 6.7%, 95% CI [0.2% – 31.9%], and DCR 26.7%, 95% CI [7.8% – 55.1%]. In a sensitivity analysis excluding patient 13 with intermediate u*MGMT*, (*n* = 14) ORR was 7.1% 95% CI [0.2% – 33.9%], DCR 21.4% 95% CI [4.7%% – 50.8%].

**Fig. 2:**
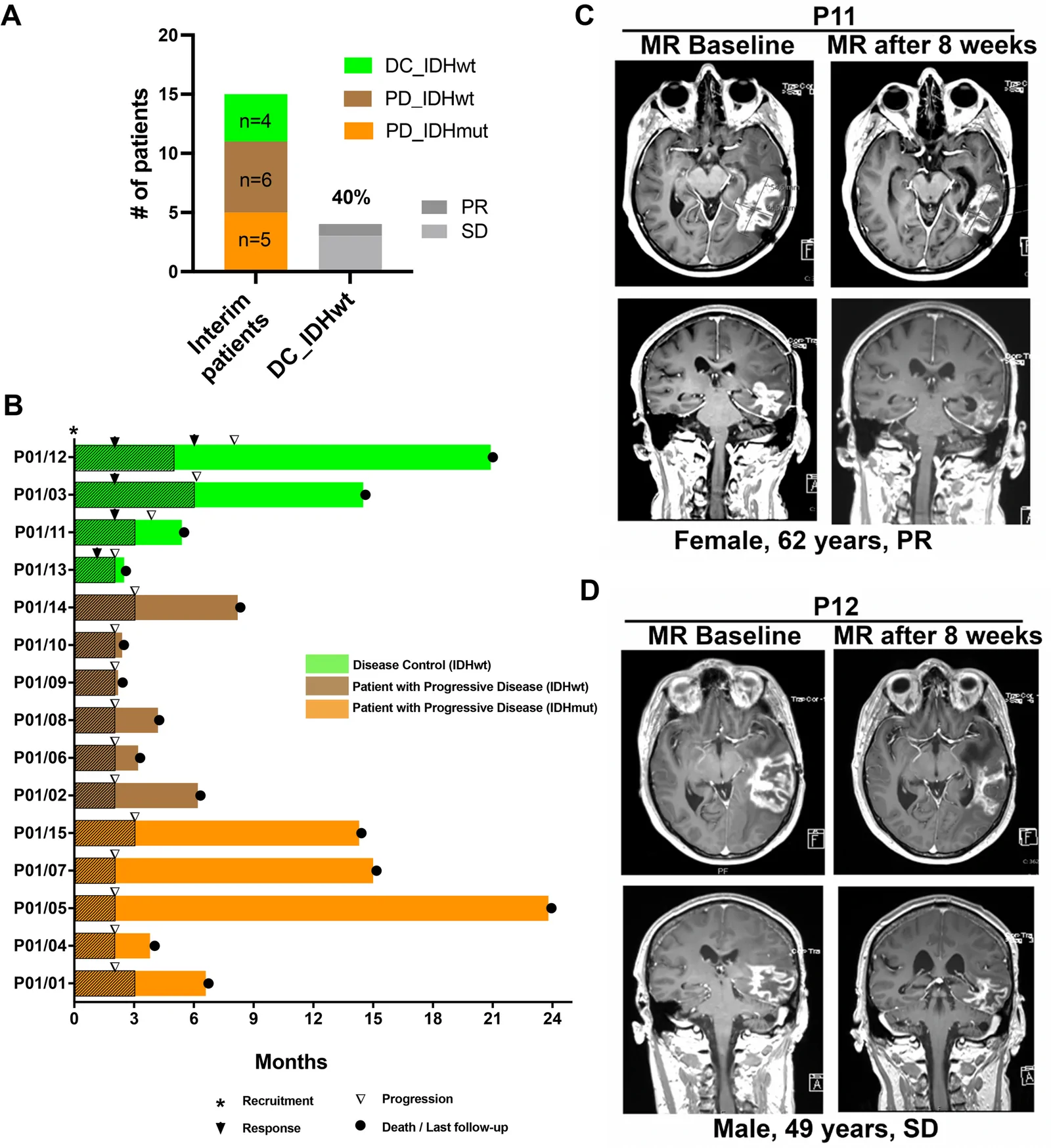
BTZ + TMZ treatment results in objective radiological response and clinical benefit. (**A**) Number of patients achieving disease control (partial response (PR, *n* = 1) and stable disease (SD, *n* = 3)), *IDH*-wildtype GBM and grade 4 *IDH*-mutant astrocytoma included in this exploratory interim analysis and (**B**) Swimmer plot showing the disease trajectories, duration of follow-up time and clinical outcomes (survival in months) for individual patients from recruitment and the number of bortezomib (BTZ) treatment cycles administered. Disease control, defined as partial response or stable disease, was achieved in four patients, all of whom had *IDH*-wildtype glioblastoma (green bars). One patient achieved a partial response, while three maintained stable disease during the treatment period. *IDH*-wildtype glioblastoma patients with progressive disease are shown in brown, and grade 4 *IDH*-mutant astrocytoma patients with progressive disease are shown in orange. Symbols indicate recruitment (asterisk), response (filled inverted triangle), progression (open inverted triangle), and death or last follow-up (filled circle), shaded area BTZ # of cycles. (**C**) representative 3D T1-weighted gadolinium contrast-enhanced serial MR images (at screening (baseline) and after 8 weeks (MRI)) of patient 11 (62-year-old female) and (**D**), patient 12 (49-year-old male)

Among patients evaluated in the interim analysis, disease control, was observed exclusively in patients with *IDH*-wildtype glioblastoma (GBM) (4/10, 40%; Fig. 2A). One patient achieved a PR, and three maintained SD throughout the treatment period. The median age of patients who achieved disease control was 54 years (range, 49–62), compared with 49 years (range, 25–58) among the remaining patients (Table 1). Median overall survival from diagnosis was 28.75 months among patients with *IDH*-wildtype GBM who achieved disease control and 16.95 months among those who experienced progressive disease during BORTEM-17 treatment (HR 0.35, 95% CI 0.09–1.43; *p* = 0.14). Patients with grade 4 *IDH*-mutant astrocytoma had a median overall survival of 28.7 months, compared with 28.75 months among patients with *IDH*-wildtype GBM who achieved disease control (HR 0.71, 95% CI 0.17–2.94; *p* = 0.64). Patients with *IDH*-wildtype GBM who experienced progressive disease during BORTEM-17 treatment had shorter overall survival than treatment-resistant patients with grade 4 *IDH*-mutant astrocytoma 16.95 vs. 28.7 months (HR 6.55, 95% CI 1.41–30.52; *p* = 0.02).

Median overall survival after recruitment was 9.95 months among patients with *IDH*-wildtype GBM who achieved disease control and 3.7 months among those who experienced progressive disease during BORTEM-17 treatment (HR 0.35, 95% CI 0.09–1.43; *p* = 0.14) reflected in the swimmer plot, Fig. 2B. Patients with grade 4 *IDH*-mutant astrocytoma had a median overall survival of 14.3 months, compared with 9.95 months among patients with *IDH*-wildtype GBM who achieved disease control (HR 0.7, 95% CI 0.17–2.90; *p* = 0.62). Patients with *IDH*-wildtype GBM who experienced progressive disease during BORTEM-17 treatment had shorter overall survival than treatment-resistant patients with grade 4 *IDH*-mutant astrocytoma (HR 5.1, 95% CI 1.18–22.04; *p* = 0.03).

PFS was defined as the interval from study enrolment to radiologically confirmed disease progression or death from any cause, whichever occurred first. TTP was defined as the interval from study enrolment to radiologically confirmed disease progression. Progression was documented in all 15 patients before death. Therefore, no observations were censored in either the PFS or TTP analysis. At 6 months after study enrollment, one of the 15 patients remained alive without documented disease progression, corresponding to an overall PFS-6 rate of 6.7% (1/15). By subgroup, PFS-6 was 25% (1/4) among patients with *IDH*-wildtype glioblastoma who achieved disease control and 0% among both patients with progressive *IDH*-wildtype glioblastoma (0/6) and patients with progressive grade 4 *IDH*-mutant astrocytoma (0/5). The patient who remained progression-free at 6 months had a PFS of 8.4 months.

Median TTP was longer among patients with *IDH*-wildtype glioblastoma who achieved disease control than among those with progressive *IDH*-wildtype glioblastoma (4.65 vs. 2.00 months). Median TTP was also longer among patients with *IDH*-wildtype glioblastoma who achieved disease control than among patients with progressive grade 4 *IDH*-mutant astrocytoma (4.65 vs. 1.90 months). Among patients with progressive disease, median TTP did not differ between those with *IDH*-wildtype glioblastoma and those with grade 4 *IDH*-mutant astrocytoma (2.00 vs. 1.90 months). A sensitivity analysis excluding patient 13, who had an intermediate *MGMT* promoter methylation status, is presented in the Supplementary Information.

Representative MR images illustrate these outcomes (Fig. 2C-D). A 62-year-old female patient (P11) (Fig. 2C) demonstrated PR with a marked 42% reduction in tumour volume compared with baseline MRI. Treatment was continued and the patient-maintained disease stability for 4 months from recruitment. Another case, a 49-year-old male patient (P12) (Fig. 2D), classified as SD according to RANO criteria, showed durable radiological stability with no significant tumour progression for over 8 months following enrollment.

Nine of the 15 patients received bevacizumab containing treatment (LAVA- combination of lomustine (100 mg/m^2^), vincristine (2 mg), and bevacizumab (400 mg day 1 and 15) (LAVA) in a 6-week cycle) post withdrawal, 1 patient who was clinically stable was re-irradiated /SRS, whilst 5 patients who had clinically deteriorated did not get further treatment (Table 1). LAVA is standard treatment at reccurence at our institution. However, some patients in phase IB were referred from other hospitals, therefore some heterogeneity in the therapy post withdrawal from study. These data indicate that BTZ + TMZ therapy yields measurable radiological response in a subset of patients, with tumour stabilization and volumetric reduction associated with clinical benefit.

### Preferential EGFR amplification and enhanced NFκB in patients achieving disease control

**Fig. 3:**
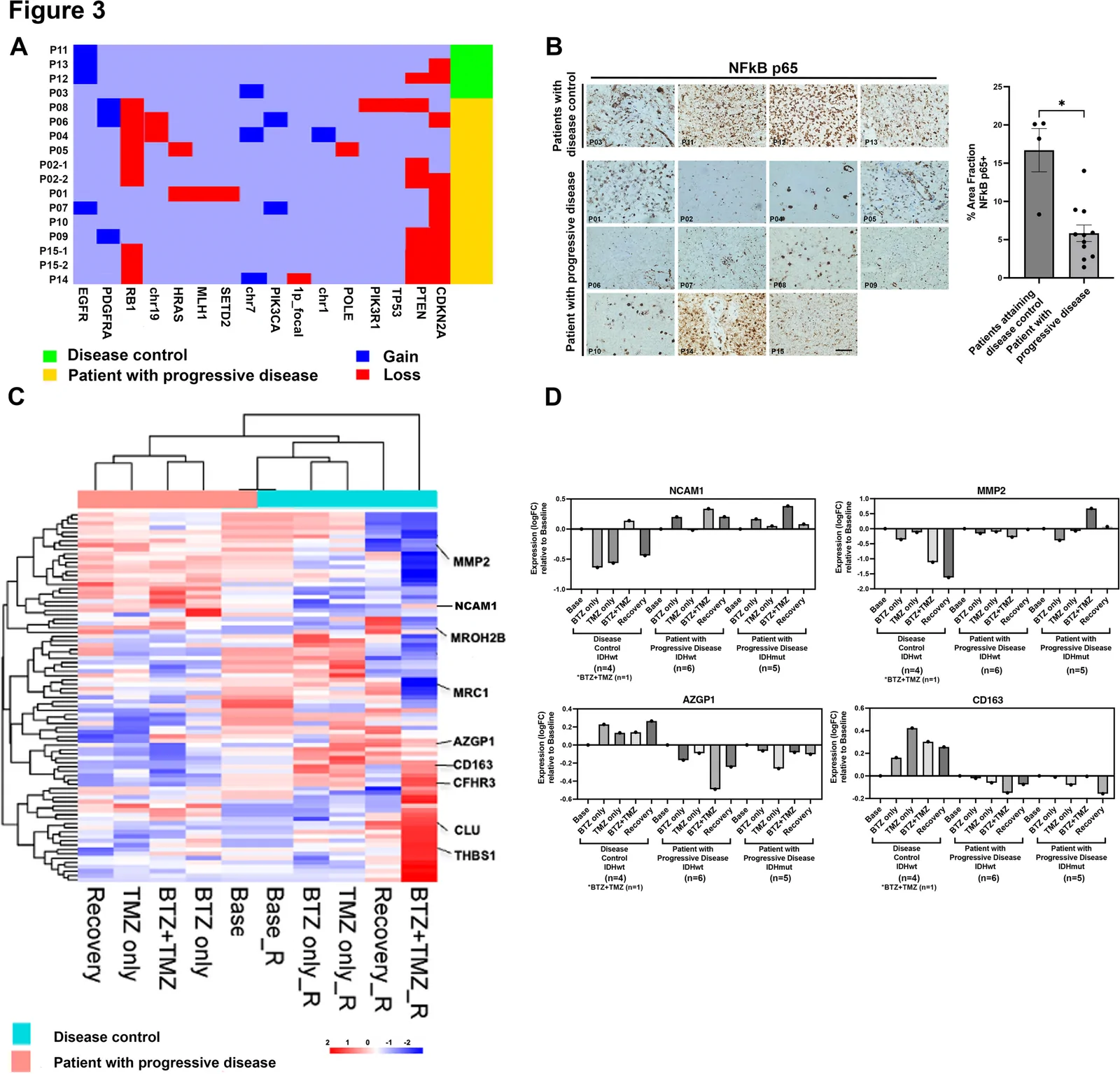
Preferential EGFR amplification and NF-κB-activation in patients achieving disease control. (**A**) Heatmap of copy number alterations identified using a targeted glioma associated gene panel for each patient; the green bar represents patients achieving disease control, and the yellow bar represents the remaining patients. For copy number alterations, blue indicates gain and red indicates loss. Patients 2 and 15 were re-operated, (**B**) Left panel: Representative immunohistochemical staining for activated, nuclear NF-κB p65 in formalin-fixed, paraffin-embedded tumour sections obtained at primary surgery. Right panel: Quantification of the percentage of NF-κB-positive nuclei. Each dot represents mean values from 4 independent regions of an individual patient tumour. Bars indicate mean ± SEM. Statistical significance was determined using an unpaired *t* test (*P < 0.05), (**C**) Heatmap of statistically significant differentially expressed cell death-related proteins (FDR < 0.05), identified by LC-MS/MS, showing log fold-change (LogFC) in plasma compared to baseline in patients achieving disease control (_**R**) and the remaining patients across treatment timepoints, where Base = Day 1 before BTZ administration; BTZ only = D1_4h + D1_24h; TMZ only = D4 + D7 before BTZ administration; BTZ + TMZ = D7_4h after BTZ administration; and Recovery = D11 + D15 + D22, (**D**) Bar plots of LogFC protein expression relative to baseline in *IDH* wild type GBM patients achieving disease control (*n* = 4) where *n* = 1 contributed sample after BTZ + TMZ, in this response group, vs. *IDH* wild type GBM with disease progression (*n* = 6) and grade 4 *IDH*-mutant astrocytoma patients (*n* = 5) with disease progression across the treatment timepoints defined in panel **C**. Only statistically significant proteins that survived Tukeys or Sdak post-hoc correction for multiple testing with are indicated

Targeted sequencing of glioma-associated genes from tumour DNA showed that three of the four *IDH*-wildtype glioblastoma patients with disease control harboured *EGFR* amplification (Fig. 3A) and only carried *PTEN* and *CDKN2A* mutations. The remaining patient exhibited gain of function of chromosome 7 without additional mutations. *EGFR* enrichment was assessed using Fisher’s exact test, revealing that *EGFR* gain was significantly more frequent in patients achieving disease control (*p* = 0.03; OR = 19.98; 95% CI [0.87–1691.98]), although the wide confidence interval reflects the small sample size.

In contrast, all patients who experienced rapid progressive disease, including all 5 grade 4 *IDH*-mutant astrocytoma, harboured alterations affecting the retinoblastoma cell cycle regulatory axis, including *CDKN2A* and/or *RB1* loss. These data suggest that advanced molecular evolution involving *CDKN2A/RB1* checkpoint disruption may override the favourable biology typically associated with *IDH*-mutant gliomas and confer resistance to sequential BTZ and TMZ. One outlier patient (P07) who harboured *IDH*-mutant astrocytoma with amplified *EGFR* and *CDKN2A* loss alongside a gain-of-function *PIK3CA*, exhibited prolonged survival, but did not achieve a radiological response according to RANO criteria. This outlier suggests that *EGFR* amplification alone is insufficient to promote response when in the presence of concurrent cell-cycle checkpoint disruption or PI3K pathway activation. Immunohistochemistry analysis of NFκB (RelA/p65) demonstrated significantly higher nuclear p65 positivity in tumours obtained at diagnostic surgery with *EGFR* amplification from patients with disease control compared with the rest of the patients (*p* < 0.05; Fig. 3B). Together, these findings may support the hypothesis that disease control is enriched in *EGFR*-amplified *IDH-*wildtype glioblastomas with preserved *RB-*pathway integrity, potentially through a signalling context permissive for NF-κB activation (Table 2).

**Table 2:** Summary of disease control and resistance mechanisms

Subgroup	Key Characteristic Alteration	Clinical Response	Hypothesized Biological Mechanism
IDH-wildtype GBM (*n* = 4)	EGFR Amplification RB1 wildtype 50% CDKN2A loss PIK3CA-wildtype NF-kB activation	**Disease control** 1 PR and 3 SD Median OS from diagnosis: 28.75 months Survival from Recruitment: 9.95 months TTP:4.65 months	Activation of EGFR/NF-kB signaling promotes MGMT expression, providing the direct molecular target for BTZ-mediated depletion and TMZ sensitization. Presence of tumor suppressor RB maintains G1/S checkpoint intact.
IDH-wildtype GBM (*n* = 6)	EGFR-wildtype 67% RB1 loss 83% CDKN2A loss PIK3CA-wildtype No NF-kB activation	**Progressive Disease** Median OS from diagnosis: 16.95 months Survival from Recruitment: 3.7 months TTP: 2.0 months	Unchecked G1/S cell cycle transition through inactivation of p16NK4A-CDK4/6-RB tumor suppressor pathway. Loss of p16 mediated inhibition permits persistent CDK4/6 activity, RB hyperphosphorylation, E2F activation and uncontrolled cell proliferation. Lack of NF-kB activation abolishes target for BTZ mediated MGMT depletion.
IDH-mutant Grade 4 Astrocytoma (*n* = 5)	EGFR-wildtype 60% RB1 loss 60% CDKN2A loss PIK3CA-wildtype No NF-kB activation	**Progressive Disease** Median OS from diagnosis: 28.7 months Survival from Recruitment: 14.3 months TTP:1.9 months	CDKN2A/RB1 cell-cycle checkpoint losses drive G1/S progression independently of MGMT-mediated DNA repair, rendering MGMT depletion futile. Acquisition of CDKN2A/B deletion, RB1 loss and PI3K pathway activation mediated cell cycle deregulation eclipses resistance to MGMT depletion and IDH mutation advantages.

Overall survival was from diagnosis to death or last observation/follow up. Survival from recruitment was recorded as time from recruitment/enrollment to death or last observation/follow-up. TTP was defined as the interval from study enrollment to radiologically confirmed disease progression. Progression was documented in all 15 patients before death. Therefore, no observations were censored in either the PFS or TTP analysis. All 15 patients were deceased at time of analysis and no observations were censored in the overall survival analysis

### Sequential BTZ and TMZ treatment is feasible with manageable hematologic and non-hematologic toxicities

The total number, type, and grade of AEs reported for each patient are provided in Supplementary Table 1. A total of 61 adverse events (AEs) were reported (Supplementary Table 2). The number and proportion of individual patients affected by each toxicity, classified according to the maximum grade experienced by each patient are presented in Table 2. Most AEs were grade 1 or 2. The most frequent non-hematological AEs were fatigue (5/15, 33.3%), constipation (4/15, 26.7%), fall (4/15, 26.7%), paresthesia (3/15, 20.0%). The principal hematological toxicity was thrombocytopenia, which affected 3/15, 20.0%. Thrombocytopenia is a recognized toxicity of bortezomib and was consistently resolved by day 22 of each treatment cycle. However, thrombocytopenia is also a known adverse effect of temozolomide. Grade 3–4 toxicities were infrequent and included thrombocytopenia, delirium, epileptic seizure, fall, hyperglycemia, suicidal ideation, suspected febrile and neutropenia. These events were considered primarily related to the underlying disease rather than to treatment. All severe treatment-related AEs resolved, spontaneously to grade 2 or less, allowing treatment to continue until progressive disease was confirmed. No grade 5 AEs occurred. Because temozolomide and bortezomib were administered in combination to sensitize GBM cells, attribution to an individual drug was not possible. Attribution was therefore based on the known toxicity profiles of temozolomide and bortezomib and on established disease-related effects, thus AEs were reported for the combination regimen rather than for each agent separately. There were no dose limiting toxicities within the 4-week assessment window. Although three patients experienced a total of nine treatment interruptions, none of these interruptions met the criteria for a DLT or resulted in dose adjustment during the DLT assessment window. Disease progression was documented in all 15 patients before death. All deaths occurred during subsequent disease follow-up, after discontinuation of study treatment and withdrawal from the study because of disease progression. In summary, combination therapy with temozolomide and bortezomib was feasible, with manageable hematological and non-hematological toxicities.

**Table 3:** Number of adverse events experienced by patients and grading of first *n* = 15 BORTEM-17 patients

	G1	G2	G3	G4	Any Grade
Abdominal pain		1/15			1/15 (6.7%)
Anal hemorrhage	1/15				1/15 (6.7%)
Anemia		1/15			1/15 (6.7%)
Anorexia		1/15			1/15 (6.7%)
Anxiety	1/15	1/15			2/15 (13.3%)
Blurred vision (right side when tired)	1/15				1/15 (6.7%)
Constipation	3/15	1/15			4/15 (26.7%)
Delirium			1/15		1/15 (6.7%)
Depression	1/15				1/15 (6.7%)
Diarrhea	1/15				1/15 (6.7%)
Dysarthria	1/15				1/15 (6.7%)
Dyspepsia	1/15				1/15 (6.7%)
Epileptic seizure			1/15		1/15 (6.7%)
Fall	3/15		1/15		4/15 (26.7%)
Fatigue		5/15			5/15 (33%)
Gait disturbance	1/15				1/15 (6.7%)
Gastrointestinal infection (suspected)	1/15				1/15 (6.7%)
Headache		2/15			2/15 (13.3)
Hyperglycemia			1/15		1/15 (6.7%)
Hypertension	1/15				1/15 (6.7%)
Hypokaliemia	1/15				1/15 (6.7%)
Hypophosphatemia		1/15			1/15 (6.7%)
Insomnia		1/15			1/15 (6.7%)
Intracranial hemorrhage	1/15				1/15 (6.7%)
Muscle weakness lower limb	1/15		1/15		2/15 (13.3%)
Nausea	1/15	1/15			2/15 (13.3%)
Paresthesia	2/15	1/15			3/15 (20%)
Platelet count decreased			1/15	2/15	3/15 (20%)
Pruitus		1/15			1/15 (6.7%)
Suicidal ideation			1/15		1/15 (6.7%)
Suspected febrile neutropenia			1/15		1/15 (6.7%)
Urinary incontinence	1/15				1/15 (6.7%)
Urinary tract infection	1/15				1/15 (6.7%)
Urticaria		1/15			1/15 (6.7%)
Vomiting	1/15				1/15 (6.7%)
No events					3/15 (20%)

Adverse events (AEs) were coded using the Medical Dictionary for Regulatory Activities (MedDRA) and graded according to the National Cancer Institute Common Terminology Criteria for Adverse Events (CTCAE), version 4.03. Data are presented as the number of distinct patients experiencing each AE at the maximum grade recorded for that patient, divided by the number of evaluable patients (n/N), with percentages shown for AEs of any grade. Each patient was counted only once per AE. All 15 treated patients were included in the safety analysis

### Proteomic profiling reveals pharmacodynamic changes in tumour aggressiveness and immune response

To investigate systemic protein changes associated with response, LC-MS/MS-based proteomic profiling of plasma collected before, during and after treatment was performed. In the longitudinal analysis of the plasma samples from the *n* = 15 patients’ before, during and after treatment, 1209 proteins were detected by LC-MS/MS and 649 differentially expressed were analyzed by multiple linear (ANOVA) and linear mixed effects models which showed differences between patient response group and treatment timepoints, (Supplementary Table 3).

Unsupervised clustering revealed coordinated protein regulation patterns across treatment phases in *IDH-*wildtype glioblastoma patients achieving disease control that were distinct from the remaining cohort (Fig. 3C). Several proteins linked to cell-adhesion and invasion, included NCAM1, and MMP2 that were downregulated in these patients (Fig. 3D), potentially suggesting reduced invasive potential. In contrast, MMP2 levels were less altered after treatment in the rapidly progressing *IDH*-mutant astrocytoma and *IDH-*wildtype glioblastoma patients harbouring *CDKN2A* and/or *RB1* loss. Proteins involved in immune modulation were also altered, including AZGP1 that suppresses proinflammatory cytokine release, polarizes macrophages towards antitumour phenotype, reduces matrix remodelling and limits tumour invasion [21] which was upregulated in patients achieving disease control. CD163 that is expressed by macrophages associated with anti-inflammatory responses, was in excess levels, as was Haptoglobin-Related Protein (HPR), a haemoglobin-binding plasma protein that is recognized by the CD163 scavenger receptor on macrophages [22], and CFHR3 were also highly upregulated in these patients. Importantly, the expression levels of these proteins were not significantly different at baseline/prior to treatment in patients achieving disease control compared to the rest of the cohort (supplementary Fig. 1A), consistent with positive within patient correlation in the longitudinal analyses. Individual patient protein abundance (Log2) trajectories throughout the treatment course are shown in supplementary Fig. 1B. Taken together, these findings suggest systemic treatment-induced proteomic changes indicative of reduced tumour aggressiveness, immune processes involved in clearance of dying cells through scavenging heme-related damage and compliment mediated inflammation.

### Proteomic profiling reveals treatment-associated changes in platelet-related processes

**Fig. 4:**
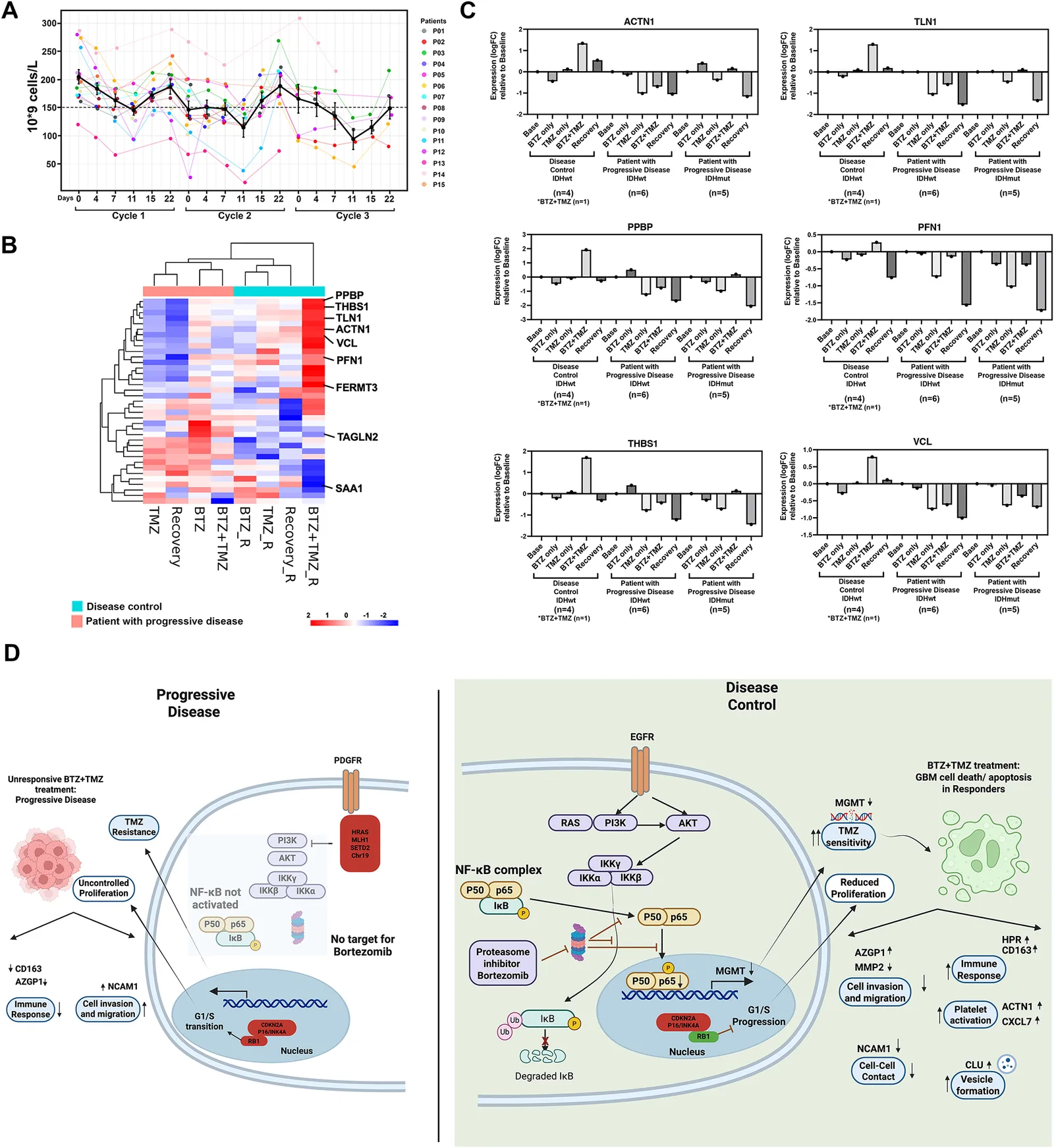
Proteomic profiling reveals treatment-associated changes in platelet-related processes. (**A**) Platelet counts (×10⁹ cells/L) measured across three treatment cycles at indicated timepoints. Each coloured line represents an individual patient. The thick black line indicates the mean platelet count across all patients at each time point ± SEM, (**B**) Heatmap of differentially expressed platelet-related proteins (FDR < 0.05), identified by LC-MS/MS, showing effect size log fold-change (LogFC) values in plasma compared to baseline in patients achieving disease control and the remaining patients across treatment timepoints, where Base = Day 1 before BTZ administration; BTZ only = D1_4h + D1_24h; TMZ only = D4 + D7 before BTZ administration; BTZ + TMZ = D7_4h after BTZ administration; and Recovery = D11 + D15 + D22, (**C**) Bar plots showing LogFC protein expression relative to baseline in *IDH* wild type GBM patients achieving disease control (*n* = 4) where *n* = 1 contributed sample after BTZ + TMZ, in this response group, vs. *IDH* wild type GBM with disease progression (*n* = 6) and grade 4 *IDH*-mutant astrocytoma patients (*n* = 5) with disease progression across the treatment timepoints defined in panel B. Only statistically significant proteins that survived Tukey’s or Sdak post-hoc correction for multiple testing with are indicated (**D**) Schematic illustration summarizing the proposed biological mechanism underlying the BTZ + TMZ treatment response or failure by disease entity in patients achieving disease control (right) and those with progressive disease (left)

Patients exhibited a reproducible pattern of platelet count fluctuation during therapy, with relative recovery between cycles, stabilization over time and normalization by day 22 of each BTZ + TMZ treatment cycle (Fig. 4A). To assess changes in platelet-associated proteins, hierarchical clustering and differential expression analyses were performed on proteomic data obtained before, during and after treatment (Fig. 4B). Distinct expression patterns were observed between *IDH-*wildtype glioblastoma patients achieving disease control and the rapidly progressing patients, with clustering driven by cytoskeletal and adhesion-related proteins. Patients achieving disease control showed less marked reduction of ACTN1 and TLN1 during treatment and recovery these proteins during treatment, while they downregulated in the rest of the cohort (Fig. 4C). PPBP (CXCL7), THBS1 and VCL were also relatively preserved in patients achieving disease control, supporting role in platelet chemotaxis, enhanced aggregation and cytoskeletal organisation, where decline in PFN1 during recovery, might suggest altered cytoskeletal remodelling. Overall, patients achieving disease control exhibited less pronounced decrease of platelet related proteins following treatment, suggesting maintenance of systemic physiologic function during therapeutic response.

## Discussion

Treatment options for *IDH-*wildtype glioblastoma patients with recurrent unmethylated *MGMT* remain limited where TMZ is established to have limited efficacy [13, 20]. There is emerging uncertainty regarding the optimal treatment for newly diagnosed grade 4 *IDH*-mutant astrocytoma, where vorasidenib in combination with TMZ, or safusidenib is under investigation in several clinical trials [23]. Regardless treatment at first diagnosis, recurrent disease is associated with poor prognosis and no universally accepted standard systemic therapy is established for either disease entity. Lomustine and bevacizumab remain among the most extensively studied agents as last-line treatment for recurrent *IDH-*wildtype glioblastoma, but they confer only modest improvements in progression-free or overall survival and rarely produce durable responses [24]. Early indications are that upon disease recurrence, vorasidenib may garner limited benefit in grade 4 *IDH*-mutant astrocytoma [25]. Our NCT03643549 phase IB/II trial initiated in 2018, investigated whether overcoming *MGMT*-mediated temozolomide resistance may resolve an important unmet clinical need.

This interim analysis of the first 15 patients enrolled in the BORTEM-17 phase IB/II trial demonstrates that sequential treatment with proteasome inhibitor BTZ followed by TMZ had a manageable toxicity profile and biologically active in a subset of recurrent *IDH-*wildtype glioblastoma patients with unmethylated *MGMT* promoter. The regimen resulted in disease control in 4/10 of these patients, including one partial response and three cases of stable disease, thereby meeting the prespecified threshold for continuation of the study. All patients were recruited based on standard methylation specific PCR (MSP) for *MGMT* methylation status and quantitative pyrosequencing was used to verify levels. P13 had unmethylated *MGMT* on MSP but intermediate values on pyrosequencing. Although stable disease after 2 treatment cycles had brief duration, protocol criteria for response assessment had been fulfilled. Strikingly, all five patients with grade 4 *IDH*-mutant astrocytoma, experienced rapid progressive disease. This distinction is particularly relevant because the trial was initiated in 2018, before the current WHO CNS5 classification [1] formally separated glioblastoma, *IDH*-wildtype from grade 4 *IDH*-mutant astrocytoma as distinct disease entities. Three of four patients achieving disease control harboured amplified *EGFR*, but all four had nuclear activation of NF-κB p65/RelA and had absence of *RB1* pathway disruption. This association is biologically plausible as *EGFR* amplification occurs in a substantial proportion of *IDH*-wildtype glioblastomas and drives receptor tyrosine kinase signalling through multiple downstream survival pathways, including PI3K-AKT, MAPK, STAT3, and NF-κB. The latter promotes pro-survival transcriptional programmes and binds to *MGMT* promoter, where abrogation results in resistance to alkylating therapy [26]. Therefore, tumours with gain of function/amplified *EGFR* and active NF-κB signalling may be particularly dependent on proteasome-regulated adaptive survival mechanisms. In this setting, BTZ may suppress NF-κB-driven survival signalling and MGMT expression, thereby sensitizing tumour cells to TMZ-induced DNA damage. This interpretation is supported by the proteomic findings, where patients achieving disease control showed comparatively attenuated treatment-induced changes in proteins involved in invasion and extracellular matrix remodelling, while proteins associated with immune mediated damage clearance, physiological function was maintained in contrast to patients with progressing disease. This pattern suggests that response to BTZ and TMZ may involve both tumour-intrinsic and host systemic pharmacodynamic effects, where responder plasma proteome might reflect not only tumour cell death, but host ability to process the treatment-induced tumour injury.

The most biologically striking result, however, was the uniform treatment failure observed among the five patients with grade 4 *IDH*-mutant astrocytoma. This finding might appear paradoxical in light of the established clinical observation of better prognosis of grade 4 *IDH*-mutant astrocytoma compared to *IDH*-wildtype glioblastomas. However, our findings are corroborated by recent results from the CATNON trial [10], that demonstrated that *IDH*-mutant patients harbouring *CDKN2A/B* loss had worse outcome. All five recurrent grade 4 *IDH*-mutant tumours in the present cohort had acquired high-risk alterations affecting the p16-CDK4/6-RB cell-cycle checkpoint axis. Specifically, they harboured *CDKN2A* and/or *RB1* loss, with one patient also demonstrating *PIK3CA* gain. These alterations are strongly associated with malignant progression in *IDH*-mutant astrocytoma where homozygous *CDKN2A/B* deletion is now incorporated into the WHO CNS5 criteria as sufficient for assignment of grade 4, reflecting the adverse prognostic significance [8, 1]. More recent studies propose additional alterations involving cell-cycle and growth signalling pathways, including *CDK4*, *CCND2*, *PDGFRA*, *PIK3CA/PIK3R1*, *MYCN*, and *EGFR*, to identify higher-risk subsets within *IDH*-mutant astrocytoma [2]. *IDH* mutation is an early lineage-defining event that generates D-2-hydroxyglutarate and induces widespread epigenetic, metabolic, DNA repair, and immune microenvironmental changes [27, 28]. These features may contribute to the relatively favourable trajectory of many *IDH*-mutant astrocytomas [28–33], however, with recurrence and malignant progression, additional molecular lesions can emerge due to clonal selection, effect of prior therapy, and genomic instability [2, 8]. Once alterations such as *CDKN2A/B* deletion or *RB1* loss are acquired, the biology of the tumour may become dominated by cell-cycle deregulation.

Mechanistically, loss of *CDKN2A/B* removes p16INK4A-mediated inhibition of CDK4/6, resulting in RB phosphorylation, E2F activation, and unchecked G1/S transition [34, 35]. *RB1* loss disables this checkpoint more directly, allowing cell-cycle progression to proceed despite cellular stress or DNA damage [36, 37]. In the context of BTZ and TMZ treatment, such alterations may reduce the therapeutic impact of MGMT depletion by permitting continued proliferation despite TMZ-induced DNA damage. Thus, even if BTZ successfully suppresses MGMT expression, tumour cells with collapsed RB-mediated checkpoint control may be able to bypass the intended cytotoxic bottleneck. Although this interpretation is hypothesis-generating, it offers a plausible explanation for why all *IDH*-mutant grade 4 astrocytomas in this interim cohort progressed rapidly.

The contrast between the responding *IDH-*wildtype glioblastomas and the progressing *IDH*-mutant grade 4 astrocytomas suggests that *IDH* status alone may be insufficient as a predictive biomarker in recurrent grade 4 glioma. Instead, therapeutic response may depend on the interaction between proteasome-dependent survival signalling through receptor tyrosine kinase activation and NF-κB activity, as well as integrity of the RB-cell-cycle checkpoint axis. In the *IDH*-wildtype responders, amplified *EGFR* and nuclear NF-κB activation may identify tumours that remain dependent on BTZ-sensitive proteasome-regulated survival networks. In contrast, the *IDH*-mutant and *IDH*-wildtype non-responders may represent a molecularly advanced subgroup in which secondary cell-cycle checkpoint disruption supersedes the expected benefit of TMZ sensitivity.

Although pseudoprogression can be challenging to exclude in patients treated in recurrent glioma trials, several features support true tumour progression at study entry. Patients were enrolled at least 12 weeks after completion of radiotherapy, and the first 15 patients entered the study a median of 16 (range 8-21.3) months after primary treatment. They also exhibited clinical deterioration consistent with progressive disease and had measurable recurrent lesions located outside the original Gamma Knife irradiation margins. Taken together, these factors support true tumour progression at study entry. The safety profile of sequential BTZ and TMZ was feasible, particularly considering that all patients had previously received TMZ. The most frequent non-hematological AEs were fatigue, constipation, falls and paresthesia, whereas the principal hematological toxicity was thrombocytopenia. The latter is a common AE in patients treated with temozolomide and the doses applied in the trial are similar to those commonly used in everyday practice. Moreover, platelet nadirs followed a predictable cyclical pattern, with recovery before subsequent treatment cycles, thereby allowing treatment to continue. Grade 3–4 toxicities were infrequent and considered primarily related to the underlying patient related conditions rather than to treatment. No treatment-related deaths occurred. If the predefined hematological criteria were not met at scheduled timepoint for the following chemotherapy cycle, the treatment was postponed for up to 2 weeks. Treatment interruption for longer than 2 weeks indicated therapy termination. However, no patient had to discontinue the treatment due to toxicity. This underscores that the treatment is feasible, but regular assessment of toxicity is mandatory.

The observation that patients achieving disease control received a lower median cumulative TMZ dose per cycle is consistent with our sensitization hypothesis whereby BTZ enhances tumour susceptibility to TMZ, reducing the TMZ dose required to achieve therapeutic effect. This aligns with our preclinical findings [15, 17] showing that BTZ inhibits NF-κB activation, depletes MGMT protein, disrupts pro-survival autophagic flux, increases DNA damage and synergistically sensitizes unmethylated *MGMT* glioblastoma cells to TMZ. In patient-derived xenograft models, BTZ was detectable in brain tissue ex vivo and prolonged survival. These data support a mechanism whereby BTZ impairs DNA repair and survival signalling, rendering TMZ activity less dependent on cumulative TMZ dose exposure.

The importance of schedule and pharmacodynamic sequence is underscored by the negative phase III results with marizomib, an irreversible proteasome inhibitor, when added to standard therapy [38]. That trial suggests that proteasome inhibition per se is not sufficient to improve outcome in an unselected glioblastoma population. The sequential dose and timing schedule in our study [19], in which BTZ precedes TMZ, is mechanistically distinct from prior negative trials because it was developed specifically to deplete MGMT and disrupt adaptive survival pathways before alkylating DNA damage is induced. Thus, the timing, reversibility, dose intensity, and molecular biological context of proteasome inhibition may be critical determinants of therapeutic activity. The platelet-associated proteomic signature observed in patients with disease control may provide an additional pharmacodynamic indicator of treatment response. Both TMZ and BTZ can induce transient thrombocytopenia, and the recovery of platelet counts between treatment cycles suggests a cyclical and self-limiting haematological effect rather than cumulative bone marrow toxicity [39]. Patients who achieved disease control exhibited a smaller treatment-induced decline in plasma levels of several platelet-associated proteins, including ACTN1, TLN1, PFN1, THBS1, VCL, and PPBP/CXCL7, compared with patients with progressive disease.

These proteins are involved in cytoskeletal remodelling, including ACTN1 and VCL; adhesion, including TLN1 and THBS1; and platelet activation, secretion, and degranulation, including PFN1 and PPBP/CXCL7 [40–42]. The reative preservation of these proteins may reflect platelet activation or turnover, or a vascular stress response associated with proteasome inhibition [43]. Notably, patients without disease control did not show a comparable platelet-associated proteomic response. Nevertheless, platelet-related signals may also reflect treatment toxicity or exposure, as well as differences in sample availability or patient attrition between the biological subgroups, rather than tumour response itself. Whether this proteomic signature represents treatment exposure, a host response, vascular remodelling, immune activation, or a surrogate marker of tumour sensitivity requires validation in the full trial cohort and in future studies.

Several limitations should be acknowledged. First, the sample size is small, as expected for a prespecified interim analysis, and the biomarker findings should therefore be interpreted as exploratory and hypothesis-generating. Second, although the observed molecular associations are biologically plausible, causal relationships cannot be established from this dataset. Third, plasma proteomic profiling provides an accessible longitudinal pharmacodynamic readout, but it cannot substitute for direct intratumoral assessment. Fresh post-treatment tumour tissue was not available because patients were not surgical candidates at progression, precluding direct measurement of intratumoral BTZ concentration, MGMT depletion, proteasome inhibition, NF-κB modulation, or DNA damage response at the site of disease. Despite these limitations, the interim findings provide an important early signal of clinical and biological activity. They support continued prospective evaluation of sequential BTZ and TMZ in the full trial cohort and suggest that future analyses should incorporate contemporary molecular classification, *EGFR* amplification, NF-κB activation, *CDKN2A/B* status and *RB1*pathway integrity. Whole-exome sequencing and integrated proteogenomic analysis of the full cohort will be critical to validate these candidate biomarkers and determine whether disease control is reproducibly enriched in tumours with amplified RTK/NF-κB signalling and intact RB-cell-cycle checkpoint control.

In conclusion, sequential BTZ followed by TMZ demonstrated manageable toxicity and met the prespecified interim efficacy threshold in recurrent unmethylated *MGMT* grade 4 gliomas. Disease control was observed in a subset of *IDH*-wildtype glioblastoma, characterized by *EGFR* amplification, nuclear NF-κB activation, and absence of *RB-*pathway disruption. In contrast, all patients with grade 4 astrocytoma, *IDH*-mutant and *IDH*-wildtype glioblastoma who experienced rapid progression, harboured high-risk alterations affecting the *CDKN2A/RB1* cell-cycle checkpoint axis. These findings suggest that the therapeutic benefit of BTZ-mediated TMZ sensitization may depend not only on MGMT suppression, but also on the broader molecular context of proteasome-dependent survival signalling and cell-cycle checkpoint integrity. If confirmed in the full phase II cohort, these data may enable biomarker-guided selection of patients most likely to benefit from sequential BTZ and TMZ and refine treatment strategies for recurrent unmethylated *MGMT IDH*-wildtype glioblastoma.

## Methods

Detailed methods are available in the supplementary materials.

### Study design, ethics approval declaration and participants

This was a multi-centre, open-label phase IB/II trial, registered on 2018-08-21 as BORTEM-17 ClinicalTrials.gov ID NCT03643549, however for interim analysis only data from one center were included. The study was performed in accordance with the principles of the Declaration of Helsinki. The study protocol was approved by the Regional Committee for Medical and Health Research Ethics for Western Norway, (Regionale komiteer for medisinsk og helsefaglig forskningsetikk (REK), Vest-Norge (REK Vest)) (2017/2084) on 18.12.2017 and by The Norwegian Medical Products Agency (DMP; formerly the Norwegian Medicines Agency) (17/17445-17). Further details on inclusion/exclusion criteria are available at: https://clinicaltrials.gov/ct2/show/NCT03643549?term=Bortezomib+and+temozolomide&cond=GBM&draw=2&rank=1. The sample size was calculated to be *n* = 63 intervention patients compared to at least *n* = 63 contemporary controls, based on the overall study hypothesis at significance level of 5% and statistical power of 80% and recruitment commenced 2018-08-30. Written informed consent was obtained from all individual participants. Eligible patients had histologically confirmed recurrent *IDH*-wildtype GBM or grade 4 *IDH* mutant astrocytoma with unmethylated *MGMT* promoter, measurable disease according to RANO criteria, radiographic progression ≥ 12 weeks after radiotherapy, Karnofsky performance status (KPS) ≥ 70, and adequate organ function. Key exclusion criteria included hypersensitivity to BTZ, boron, or mannitol, prior treatment with bevacizumab or lomustine (alone or as a combination) for recurrent disease, pregnancy or breast feeding. Ten patients were included in phase IB and an additional *n* = 53 were planned for inclusion in phase II. Objective radiological response was defined by the response assessment in neuro-oncology criteria (RANO) [39]. The first 15 evaluable patients (*n* = 10 males and *n* = 5 females) were included in the interim analysis. Patients were enrolled between September 2018 and June 2021, and their characteristics are summarized in Table 1.

### Simon’s MinMax-like design

Determining the number of patients and responses needed to fulfil criteria of interim utility was guided by Simon’s MinMax -like design [40, 41] where the *p0* null hypothesis was that the true response rate would be 0 and the *p1* hypothesis was that the true response rate would be 0.15. If 2 or more responses were achieved among these 15 patients, we would reject the null hypothesis and consider the response signal promising to warrant full recruitment in phase II. The design controls the type I error rate at *p*0 = 0.05 and statistical power of approximately 64.9% https://cancer.unc.edu/biostatistics/program/ivanova/SimonsTwoStageDesign.aspx.

### MGMT methylation status

*MGMT* promoter methylation status was determined from tumour DNA following bisulfite conversion, using Methylation-Specific PCR (MSP) in the pathology departments at the treating centres as part of standard diagnostic procedure. For quantitative assessment, Pyrosequencing (PSQ) was performed at the Department of Neuropathology, University of Bonn, Germany as previously described [42]. *MGMT* methylation levels were below published best-practice cutoff values of < 10–21% [ 43–45].

### Treatment regimen and endpoints

BTZ was administered intravenously at 1.3 mg/m² on days 1, 4, and 7 of a 28-day cycle. TMZ was administered orally at 150–200 mg/m² once daily on days 3–7 of the same cycle. In phase IB of the study, dose escalation of TMZ was performed: The first cohort of 3 patients received 150 mg/m² of TMZ for 5 days every 4 weeks. Dose-limiting toxicity (DLT) was defined as treatment-related toxicity effects of unacceptable severity or irreversibility and that limit further dose escalation, occurring during the first treatment cycle and graded according to National Cancer Institute Common Terminology Criteria for Adverse Events (CTCAE) version 4.03. The maximum tolerated dose (MTD) was defined as the lowest dose level at which ≥ 33% of patients experience DLT. If one patient developed a DLT, an additional cohort of 3 patients was treated at the same dose level. Dose escalation proceeded to a maximum dose of 200mg/m^2^ unless two or more patients in a cohort of 3–6 experienced DLT. Treatment continued until disease progression or unacceptable toxicity and dose reductions were allowed. The primary endpoints for phase IB were safety and toxicity. Phase II endpoints included median overall survival (OS), 6-months progression-free survival (PFS), and time to progression (TTP). The final efficacy will be reported in fully accrued phase II cohort. Secondary endpoints involved identifying novel tumour biomarkers through analyses of physiological, molecular, and biochemical changes in blood and tumour tissue that correlate with treatment responses.

### Monitoring adverse events (AEs)

Adverse events (AEs) and serious adverse events (SAEs) were monitored and graded according to International Council for Harmonisation - Good Clinical Practice ICH-GCP guidelines and graded using CTCAE version 4.03. Adverse events were reported in accordance with the CONSORT Harms 2022 recommendations [46]. Safety assessments were performed before each following cycle, i.e. every 4th week and included physical examination, neurological evaluation (NANO criteria), Karnofsky performance status, vital signs, and laboratory analyses (haematology, biochemistry, and coagulation parameters) at each treatment cycle visit. Patients were instructed to report any new or worsening symptoms. Investigators differentiated between treatment-related neurological toxicity and disease progression. Expected AEs included hematologic toxicity (thrombocytopenia, neutropenia), gastrointestinal symptoms, hepatic enzyme elevation, hyponatremia, musculoskeletal pain and peripheral neuropathy. All AEs were documented in electronic case report forms (Viedoc™, Viedoc Technologies AB, Uppsala Sweden), monitored until resolution or stabilization, and formally reported. Safety monitoring continued for 30 days after the last dose of study medication. If the patient did not meet the treatment requirement due to prolonged toxicity, the treatment would be postponed with new assessment at least weekly. Absence of recovery of hematological or biochemical toxicity 14 days after planned treatment would indicate therapy discontinuation and study withdrawal. Hematologic tests were obtained on days 1, 4, 7, 11, 15, 22, and 28 of each cycle.

### Radiological response assessment by neuro imaging

Magnetic resonance imaging (MRI) scans were performed using a 3T Siemens MAGNETOM Prisma (Siemens Healthineers, Munich, Germany) at the Radiology Department at Haukeland University Hospital. Objective radiological response was assessed using brain MRI according to RANO criteria [39] using as baseline, MRI obtained at least 12 weeks after completion of last radiotherapy. Follow-up MRIs were performed every 8–12 weeks or earlier if clinically indicated, using T1 and T2 MRI sequences with and without gadolinium contrast agent (Clariscan; GE Healthcare), and scans were evaluated by the study neuroradiologists. Objective radiological response was defined as CR or PR. Stable disease was not considered an objective radiological response but was recorded as disease control. Patients achieving disease control (PR + SD) after completing at least 2 treatment cycles were evaluable for response, analysed and compared to remaining cohort. Clinical deterioration based on NANO score or decline in KPS prompted extraordinary MRI to confirm the suspected progression.

### Molecular and pharmacodynamic biomarker signature analysis

Targeted sequencing of glioma-associated genes (Glioma Panel V2, detailed in supplementary materials) was performed using AmpliSeq for Illumina^®^ chemistry. Genomic DNA was extracted from formalin-fixed, paraffin-embedded glioblastoma (GBM) tissue blocks by using QIAmp^®^ DNA FFPE Advanced kit (56604, Qiagen, Hilden, Germany). Direct sequencing (SBS sequencing by synthesis) was performed with the Illumina v3 kit in an Illumina NGS instrument (MiSeq). Sequence data were analyzed using Variant Studio analysis software and the Integrated Genome Viewer (IGV) 2.3.68. Assessment of pathogenic relevance was evaluated using the ClinVar, SIFT, PolyPhen, Varsome, and COSMIC databases. Determination of copy number alterations (CAN) was done using the OncoCNV caller v1.2.0 [47] and Nexus 8.0 analysis software.

LC-MS/MS was used to identify novel biomarkers that correlate with objective treatment responses by determining proteomic changes in plasma collected on days 1, 4, 7, 11, 15, and 22 during the first cycle of treatment (Supplementary Table 4). Due to frequency of the planned visits, the phase II patients P11-P15 were not sampled at Day 1 (24-hour), Day 7, Day 7 (4-hour), Day 11 and Day 15, wherein among patients achieving disease control (P03; P11-13) only P03 contributed a sample to the BTZ + TMZ timepoint. For data analysis, Day 1 (4-hour) and Day 1 (24-hour) samples were used as BTZ only samples, Day 4 samples from P11-15 and D4 + D7 from P01-10 were used as TMZ only samples Day 7 (4-hour) samples used as BTZ + TMZ samples and Day 11, 15 and 22 samples from P01-10 and Day 22 samples from P11-15 were used as Recovery samples. Detailed methods are provided in supplementary materials. Briefly, the proteins in plasma samples of patients were analysed by Electrospray ionization LC-MS. Plasma samples were run on an Ultimate 3000 RSLC nano system (Thermo Scientific, Sunnyvale, California, United States) connected online to a Orbitrap Exploris 480 mass spectrometer (Thermo Scientific, Bremen, Germany) equipped with EASY-spray nano-electrospray ion source (Thermo Scientific). MS spectra (from m/z 375–1500) were acquired in the Orbitrap with resolution *R* = 60,000 at m/z 200, automatic gain control (AGC) target of 3 × 10⁶ and a maximum injection time (IT) set to Auto. MS raw files were analysed by the Proteome Discoverer software (Version 2.8), and peak lists were searched against the human SwissProt FASTA database (version May 2019), and a common contaminants database downloaded from the Global Proteome Machine in 2019. Correlation between all sample pairs based on TMT protein intensity analyses, (Supplementary Table 5) was performed in Proteome Discoverer using Perseus (v. 2.0.7.0) [48].

### Statistical and pathway analyses

For LC-MS/MS data were analysed using Perseus v2.0.3.0 to identify significant Differentially Expressed Proteins (DEPs), detailed method in supplementary materials. Default settings, (Euclidian and average linkage) were applied, and unsupervised hierarchical clustering was performed on the whole dataset. For multiple comparisons, ANOVA with permutation-based FDR (0.05) was performed in Perseus, followed by Tukeys post hoc testing in SPSS Inc. (version 25, IL, USA). Linear mixed effects models were performed for each protein. Responders and non-responders were compared in one model, while differences between treatment timepoint were set up in a separate model. For the treatment timepoint model, Sidak adjustment of p-values was performed to account for multiple comparisons of the treatment timepoints. For the linear mixed models, patient was entered as a random factor (i.e. random intercept model), while the indicator for responders versus non-responders and the categorical variable for treatment timepoint was entered as fixed effects accordingly. As an addendum we calculated Sidak adjustment for the overall p-values, adjusting these for the number of proteins analyzed. Ordinal linear models are also provided so that deviations between the ordinary linear models and the mixed linear models can be examined. Furthermore, intra class correlations (ICC, for the correlation between observations from the same individual), and design effect was calculated and presented so that the importance of choosing a mixed model over an ordinary linear model can be quantified. Effect estimates and uncertainties are provided in Supplementary Table 3. Analyses were performed using Stata version 19 (Stata corp, TX, USA). Only proteins that retained statistical significance after correction for multiple testing and distinguished response group and/or treatment timepoints were visualized in the graphs. Pathway enrichment and functional annotation were conducted using ClueGO v2.5.7, within Cytoscape v3.8.2 [49]. analyses of these DEPs, to explore associated biological processes and pathways. This analysis enabled the identification and visualization of functionally grouped significant Gene Ontology (GO), GO (Biological Process) terms, KEGG (Kyoto Encyclopaedia of Genes and Genomes), and Reactome pathways. ClueGO analysis was carried out using default parameters. *P*-values were adjusted for multiple testing using the Bonferroni step-down method and GO terms or pathways with an adjusted p-value (False Discovery Rate, FDR < 0.05) were considered significant. Heatmaps were generated using SRplot [50] to illustrate the expression patterns of the statistically significant DEPs across patient groups during different treatment timepoints. Survival probabilities for time to progression, progression free survival at 6 months, time to death from diagnosis and from recruitment were calculated using Kaplan-Meier analysis with log-rank tests for multiple group comparisons (Stata corp). 95% confidence intervals for ORR and DCR was calculated using exact binomial probabilities.

## Supplementary Information

Below is the link to the electronic supplementary material.

Supplementary Material 1

Supplementary Material 2

Supplementary Material 3

Supplementary Material 4

Supplementary Material 5

Supplementary Material 6

Supplementary Material 7

Supplementary Material 8

## Data Availability

The authors declare that the data that support the findings of this study are sensitive, thus not yet publicly available due to patient privacy protection requirements and ethical restrictions imposed by the Regional Ethics Committee in Western Norway (ref. 2017/2084/REK vest). Data are available upon reasonable request from the corresponding author (MC) for researchers who meet criteria for access to confidential data.

## Abbreviations

ACTN1: Actinin alpha 1
ACVR1: Activin A receptor type 1
AEs: Adverse events
ANOVA: Analysis of variance
ATP: Adenosine triphosphate
ATRX: Alpha-thalassemia/mental retardation X-linked
AZGP1: Alpha-2-glycoprotein 1, zinc-binding
BCL-2: B-cell lymphoma 2
BORTEM-17: Sequential bortezomib and temozolomide trial in recurrent grade 4 glioma
BRAF: B-Raf proto-oncogene, serine/threonine kinase
BTZ: Bortezomib
CAN: Copy number alteration
CATNON: Concurrent and adjuvant temozolomide for non-co-deleted anaplastic glioma
CCND2: Cyclin D2
CD163: Cluster of differentiation 163
CDK4/6: Cyclin-dependent kinases 4 and 6
CDKN2A: Cyclin-dependent kinase inhibitor 2A
CDKN2A/B: Cyclin-dependent kinase inhibitor 2A and 2B
CFHR3: Complement factor H-related 3
CI: Confidence intervals
CIC: Capicua transcriptional repressor
CNS: Central nervous system
CONSORT: Consolidated standards of reporting trials
COVID: Corona virus disease
CR: Complete response
CTCAE: National cancer institute common terminology criteria for adverse events
CXCL7: Chemokine (C-X-C motif) ligand 7
DCR: Disease control rate
DEPs: Differentially expressed proteins
DLT: Dose limiting toxicity
DMP: Direktoratet for medisinske produkter (The Norwegian medical product agency)
DNA: Deoxyribonucleic acid
E2F: E2 transcription factor
EGFR: Epidermal growth factor receptor
ESMO: The European society for medical oncology
ERBB2: Erb-B2 receptor tyrosine kinase 2
FDR: False discovery rate
FFPE: Formalin-fixed paraffin-embedded
FGFR1: Fibroblast growth factor receptor 1
FUBP1: Far upstream element-binding protein 1
GBM: Glioblastoma
GNA11: G protein subunit alpha 11
GNAQ: G protein subunit alpha Q
GO: Gene ontology
GTR: Gross total resection
H3F3A: H3 histone family member 3A
Hist1H3B: Histone cluster 1, H3 histone family, member B
Hist1H3C: Histone cluster 1, H3 histone family, member C
HPR: Haptoglobin-related protein
HR: Hazard ratio
HRAS: HRas proto-oncogene, GTPase
ICC: Intraclass correlation coefficient
IDH: Isocitrate dehydrogenase
IGV: Integrative genome viewer
IHC: Immunohistochemistry
IHC-GCP: International council for harmonisation - good clinical practice
IR: Ionizing radiation
ISH: In situ hybridization
KEGG: Kyoto encyclopedia of genes and genomes
KPS: Karnofsky performance status
LAVA: Combination of lomustine (100 mg/m^2^), vincristine (2 mg), and bevacizumab (400 mg)Liquid chromatography-tandem mass spectrometry
LogFC: Log fold-change
MAPK: Mitogen-activated protein kinase
MGMT: O^6^-methylguanine-DNA methyltransferase
MLH1: MutL protein homolog 1
MMP2: Matrix metalloproteinase-2
MRI: Magnetic resonance imaging
mRNA: Messenger ribonucleic acid
MSH2: MutS homolog 2
MSH6: MutS homolog 6
MSP: Methylation specific PCR
MTD: Maximum tolerated dose
Mut: Mutated
MYB: MYB proto-oncogene, transcription factor
MYBL1: MYB proto-oncogene like 1
MYC: MYC proto-oncogene, bHLH transcription factor
MYCN: N-myc proto-oncogene protein
NANO: Neurologic assessment in neuro-oncology
NCAM1: Neural cell adhesion molecule 1
NF-κB: Nuclear factor kappa-light-chain-enhancer of activated B cells
NF1: Neurofibromin 1
NF2: Neurofibromin 2
NGS: Next generation sequencing
NRAS: NRAS proto-oncogene, GTPase
ORR: Objective response rate
OS: Overall survival
PCR: Polymerase chain reaction
PCV: Combination of procarbazine, lomustine and vincristine
PDGFRA: Platelet-derived growth factor receptor alpha
PFN1: Profilin 1
PFS: Progression free survival
PFS-6mo: Progression free survival at 6 months
PI3K-AKT: Phosphoinositide 3-kinase and AKT serine/threonine kinase
PIK3CA: Phosphoinositide-3-kinase catalytic subunit alpha
PIK3R1: Phosphoinositide-3-kinase regulatory subunit 1
PMS2: PMS1 protein homolog 2
POLD1: DNA polymerase delta 1, catalytic subunit
POLE: DNA polymerase epsilon, catalytic subunit
PPBP: Pro-platelet basic protein
PPM1D: Protein phosphatase, Mg2+/Mn2+ dependent 1D
PR: Partial response
PROBE: Proteomics unit at the University of Bergen
PSQ: Pyrosequencing
PTEN: Phosphatase and tensin homolog
QoL: Quality of life
RANO: Response assessment in neuro-oncology
RB1: RB transcriptional corepressor 1
REK: Regionale komiteer for medisinsk og helsefaglig forskningsetikk (The regional committee for medical and health research ethics for western Norway)
RTK: Receptor tyrosine kinase
SAEs: Serious adverse events
SBS: Sequencing by synthesis
SD: Stable disease
SETD2: SET domain containing 2, histone lysine methyltransferase
STAT3: Signal transducer and activator of transcription 3
STR: Sub-total resection
TERTp: Telomerase reverse transcriptase promoter
THBS1: Thrombospondin 1
TLN1: Talin-1
TMT: Tandem mass tag
TMZ: Temozolomide
TP53: Tumour protein p53
TTP: Time to progression
uMGMT: Unmethylated MGMT promoter
VCL: Vinculin
WHO: World health organization
WT: Wild type
