# Supplementary material for: Contrasting molecular and pharmacodynamic signatures of response and resistance to sequential bortezomib and temozolomide in recurrent IDH-wildtype glioblastoma and grade 4 IDH-mutant astrocytoma: an exploratory interim analysis of NCT03643549 phase IB/II trial
Source: Biomark Res. 2026 Oct 27;14:148. doi: 10.1186/s40364-026-01023-w (PMC13715206; doi:10.1186/s40364-026-01023-w)

# Supplementary Figure 2

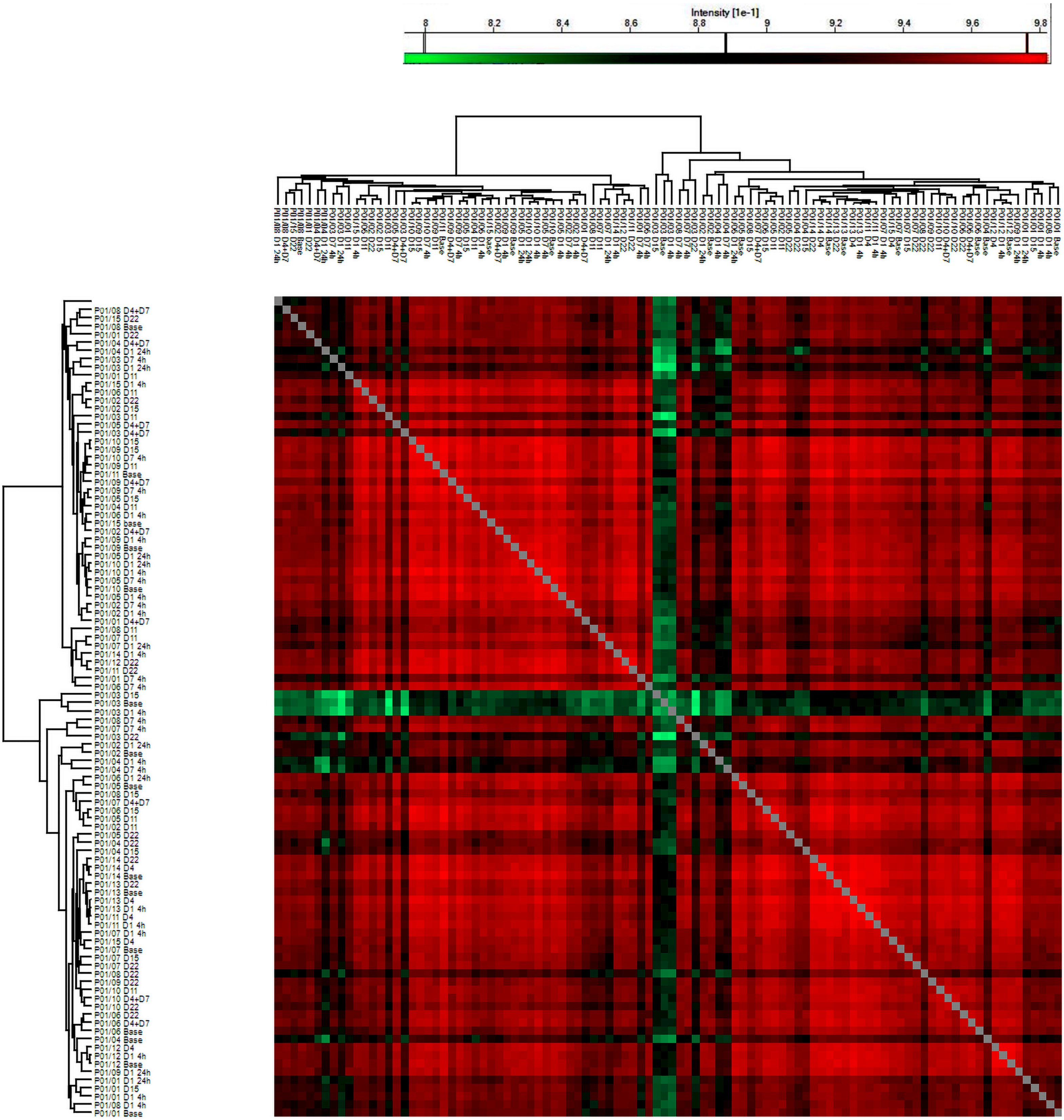

Supplement: Supplementary file 1 [file 40364_2026_1023_MOESM3_ESM.pdf]
