# Supplementary material for: Contrasting molecular and pharmacodynamic signatures of response and resistance to sequential bortezomib and temozolomide in recurrent IDH-wildtype glioblastoma and grade 4 IDH-mutant astrocytoma: an exploratory interim analysis of NCT03643549 phase IB/II trial
Source: Biomark Res. 2026 Oct 27;14:148. doi: 10.1186/s40364-026-01023-w (PMC13715206; doi:10.1186/s40364-026-01023-w)
Supplement: Supplementary file 2 [file 40364_2026_1023_MOESM1_ESM.docx]

**Supplementary results**

We performed a sensitivity analysis excluding patient 13, who had an intermediate MGMT promoter methylation status. In this 14-patient analysis, median TTP was longer among patients with IDH-wildtype glioblastoma who achieved disease control than among those with progressive IDH-wildtype glioblastoma (5.8 vs. 2.00 months). Median TTP was also longer among patients with IDH-wildtype glioblastoma who achieved disease control than among patients with progressive grade 4 IDH-mutant astrocytoma (5.8 vs 1.90 months). The comparison between patients with progressive IDH-wildtype glioblastoma and those with progressive grade 4 IDH-mutant astrocytoma was unchanged (2.00 vs. 1.90 months). Overall survival from diagnosis was defined as the interval from diagnosis to death or last follow-up, whereas survival from recruitment was defined as the interval from study enrolment to death or last follow-up. All 15 patients had died by the data cut-off; therefore, no observations were censored in the overall survival analyses.

The plasma proteomic levels of were not significantly different at baseline, prior to treatment, in patients achieving disease control compared to the rest of the cohort indicating the pharmacodynamic changes might be treatment induced (supplementary Fig 1A). This is also consistent with the positive within patient correlation in the longitudinal analyses. We observed within patient correlations (i.e. intra class correlation, ICC) that may be of importance in some proteins. In 108 of the proteins, the ICC was larger than 0.05, while the design effect was larger than 1.1 for 124 of the proteins indicating that, for these proteins, an ordinary linear model should be replaced by a linear mixed effects model (Supplementary Table 3).

**Supplementary Figure 1.** (A) Baseline expression levels of selected individual proteins in patients achieving disease control and the remaining patients. (B) Individual patients’ normalized protein abundances (log2) of NCAM1, MMP2, AZGP1, CD163, ACTN1, TLN1, PPBP, PFN1, THBS1, and VCL measured longitudinally. Samples were obtained at baseline (Day 1 before BTZ administration), during BTZ-only treatment (D1_4h and D1_24h), TMZ-only treatment (D4 and D4+D7 before BT administration), combined BTZ + TMZ treatment (BTZ+TMZ = D7_4h after BTZ administration), and during recovery (D11, D15 and D22). Each data point represents an individual patient sample.

**Supplementary Figure 2. Sample correlation analysis.**

Pearson correlation coefficients were calculated between all sample pairs based on TMT-based protein intensities, following normalization performed in Proteome Discoverer, as provided by Perseus[1] (version 2.0.7.0). Samples were subsequently clustered by hierarchical clustering (average linkage) based on Pearson correlation distance, and the resulting correlation matrix is displayed as a heatmap, with colour intensity representing the degree of pairwise correlation (r), see Supplementary Table 5 for summary statistics of sample correlation analysis.

**Supplementary methods**

**Study design, ethics approval declaration and participants**

This multicenter, controlled, non‑randomized, open‑label Phase IB/II trial evaluated biological and clinical activity of bortezomib plus temozolomide in recurrent GBM with unmethylated MGMT promoter. For interim analysis only data from one centre were included. It was registered on 2018-08-21 as BORTEM-17 ClinicalTrials.gov ID NCT03643549. This study´s primary intention was to provide biological and clinical activity and was performed in line with the principles of the Declaration of Helsinki where randomization was deemed unethical given that MGMT unmethylated patients do not respond to temozolomide [2]. The study was approved by the regional ethical board for Western Norway, REKVEst (2017/2084) on 18.12.2017 and the Norwegian Medicines Agency (17/17445‐17). Further details on inclusion/exclusion criteria are summarized and available on: <https://clinicaltrials.gov/ct2/show/NCT03643549?term=Bortezomib+and+temozolomide&cond=GBM&draw=2&rank=1>. Recruitment began 30.08.2018, with a planned sample size of *n*=63 intervention patients compared to at least *n*=63 contemporary controls. All participants provided written informed consent. Eligibility required histologically confirmed recurrent GBM with unmethylated MGMT, measurable disease by RANO, progression ≥12 weeks after radiotherapy, KPS ≥70, and adequate organ function. Key exclusions were hypersensitivity to BTZ, boron, or mannitol; prior bevacizumab or lomustine for recurrence; and pregnancy or breastfeeding. 10 patients were included in phase IB and 53 in Phase II. Because the trial began in 2018, before the 2021 WHO CNS5[3] classification introduced molecular subtyping, enrollment was not limited to *IDH*–wild‑type tumors.

*MGMT Methylation specific PCR*

DNA was purified and further processed using the EpiTect Bisulfite kit (Qiagen). PCR was run with primers specific for methylated and unmethylated MGMT promoter. Positive and Negative controls were used and appeared as expected (documented in pathology reports archived in trial documentation system).

**Monitoring adverse events (AEs)**

Adverse events (AEs) and serious adverse events (SAEs) for each patient were monitored and graded according to International Council for Harmonization - Good Clinical Practice ICH-GCP guidelines and graded using National Cancer Institute Common Terminology Criteria for Adverse Events (CTCAE) version 4.03. Safety assessments included physical examination, neurological evaluation (NANO criteria), Karnofsky performance status, vital signs, and laboratory analyses (hematology, biochemistry, and coagulation parameters) at each treatment cycle. Patients were instructed to report any new or worsening symptoms, and investigators distinguished treatment-related neurological toxicity from disease progression. Expected AEs included hematologic toxicity (thrombocytopenia, neutropenia), gastrointestinal symptoms, hepatic enzyme elevation, hyponatremia, musculoskeletal pain and peripheral neuropathy. All AEs were documented in electronic case report forms (Viedoc™, Viedoc Technologies AB, Uppsala Sweden), followed until resolution or stabilization, and reported. Safety monitoring continued for 30 days after the last dose of study medication. Hematological tests were obtained within each treatment cycle on days 1, 4, 7, 11, 15, 22, and 28 before commencing the next cycle. See supplementary Table 1 for all individual patients AEs.

**Objective radiological response assessment by Neuro Imaging**

Objective radiological response was assessed with brain magnetic resonance imaging (MRI) according to the Response Assessment in Neuro-Oncology (RANO) criteria [4]. MRI was performed using the 3T Siemens MAGNETOM Prisma (Siemens Healthineers, Munich, Germany) at the Radiology Department at Haukeland University Hospital. Baseline imaging was obtained at inclusion at least 12 weeks after completion of last radiotherapy, and follow-up MRIs were performed every 8-12 weeks or earlier if clinically indicated using T2 and T1 MRI sequences with and without gadolinium contrast agent (Clariscan; GE Healthcare) and reported by the study neuroradiologists based on RANO criteria. Contrast-enhancing lesions were measured two-dimensionally, and changes were categorized as CR, PR, SD, or PD. Objective radiological response was defined as CR or PR only in this interim analysis. Stable disease was not considered an objective radiological response but was recorded as disease control. However, because patients with recurrent glioblastoma with unmethylated *MGMT* promoter have limited expected benefit from alkylating chemotherapy and short survival, endpoints capturing tumor growth control are clinically meaningful. Therefore, in addition to objective response rate (CR+PR), the study also evaluated patient attaining disease control (CR+PR+SD), along with time-to-event outcomes (PFS, OS), to characterize therapeutic activity beyond radiographic shrinkage and clinical benefit. The clinical deterioration assessed by NANO as well as decrease in Karnofsky performance status led to extraordinary MRI to confirm the suspected progression.

**Immunohistochemistry**

Formalin-fixed, paraffin-embedded (FFPE) tissue blocks for BORTEM-17 patients were obtained at the time of primary surgery and subsequent reoperations were retrieved and processed at the Department of Pathology, Haukeland University Hospital. Diagnostic immunohistochemical staining was performed on the BenchMark ULTRA automated IHC/ISH platform (Ventana Medical Systems, Roche) using Protocol #166 (P65) with the OptiView DAB IHC v6 detection system (Ventana, Ref: 760-700) at the Department of Pathology, Haukeland University Hospital. Slides were deparaffinized on board and subjected to heat-induced epitope retrieval with Cell Conditioner, followed by incubation according to the validated protocol. The primary antibody (anti-NFkB-p65 produced in rabbit, Millipore, Ref: SAB5700780, RRID: AB_3753753) was diluted 1:200 and applied manually with an incubation time of 32 minutes at 36°C. Signal detection was performed using the OptiView HQ Universal Linker and HRP Multimer system with amplification, DAB chromogen development, and copper enhancement. Slides were counterstained with Hematoxylin II and blued with Bluing Reagent according to the manufacturer’s instructions. Stained sections were examined using a Nikon Eclipse 600 microscope (Nikon Corporation, Japan). For each case, four representative fields (20x and 40x) were captured under identical exposure settings with a Nikon Digital Camera (DXM 1,200F, Nikon). Quantification nuclear staining was performed using NIS-Elements software (version 5.3). Threshold levels of pixel intensity were determined and expressed as % positive cells/area fraction at x200 magnification.

**Library preparation, NGS and data analysis**

Enrichment of genomic target regions of glioma-associated genes (Glioma Panel V2) was performed using AmpliSeq for Illumina® chemistry. Direct sequencing (SBS sequencing by synthesis) was performed with the Illumina v3 kit in an Illumina NGS instrument (MiSeq). Sequence data were analyzed using Variant Studio analysis software and the Integrated Genome Viewer (IGV) 2.3.68. Assessment of pathogenic relevance was evaluated using the ClinVar, SIFT, PolyPhen, Varsome, and COSMIC databases. Determination of copy number alterations (CAN) was done using the OncoCNV caller v1.2.0 and Nexus 8.0 analysis software. The panel covers a total of 141.687 bp and comprised glioma specific hot spot regions, the entire coding sequence of genes frequently mutated in brain cancer and SNPs located in chromosomal regions with frequent copy number alterations. Glioma-associated Hotspot-Regions investigated included *IDH1(*R132), *IDH2*(R172), *H3F3A*(K27, G34), *TERT*p (C228T c.-124C>T Chr.5:1295228C>T, C250T c.-146C>T Chr.5:1295250C>T), *Hist1H3B* (K27), *Hist1H3C* (K27), *BRAF* (V600), *FGFR1* (N546, K655, K656). Genes included with full coverage of the coding sequence were *ACVR1, ATRX, CDKN2A, CIC, EGFR, ERBB2, FUBP1, MLH1, MSH2, MSH6, NF1, PDGFRA, PIK3CA, PIK3R1, PMS2, POLD1, POLE, PPM1D, PTEN, RB1, TP53, GNA11, GNAQ, HRAS, NRAS, MYB, MYBL1, MYC, MYCN, NF2, NRAS, SETD2*. Copy Number variants investigated included combined loss 1p/19q, amplification of *EGFR-, PDGFRA, MYC, MYCN* and deletion of *CDKN2A*.

**LC-MS/MS**

Briefly, the proteins in plasma samples of the 15 patients were analysed by Electrospray ionization LC-MS. For patient sampling and sample availability see Supplementary table 4. Due to frequency of the planned visits, the phase II patients P11-P15 were not sampled at Day 1 (24-hour), Day 7, Day 7 (4-hour), Day 11 and Day 15, wherein among patients achieving disease control (P03; P11-13) only P03 contributed a sample to the BTZ+TMZ timepoint. For data analysis, Day 1 (4-hour) and Day 1 (24-hour) samples were used as BTZ only samples, Day 4 samples from P11-15 and D4+D7 from P01-10 were used as TMZ only samples, Day 7 (4-hour) samples used as BTZ+TMZ samples and Day 11, 15 and 22 samples from P01-10 and Day 22 samples from P11-15 were used as recovery samples. Plasma samples were run on an Ultimate 3000 RSLC nano system (Thermo Scientific, Sunnyvale, California, United States) connected online to a Orbitrap Exploris 480 mass spectrometer (Thermo Scientific, Bremen, Germany) equipped with EASY-spray nano-electrospray ion source (Thermo Scientific). MS spectra (from m/z 375–1500) were acquired in the Orbitrap with resolution R = 60000 at m/z 200, automatic gain control (AGC) target of 3 × 10⁶ and a maximum injection time (IT) set to Auto. MS raw files were analyzed by the Proteome Discoverer (Version 2.8) and peak lists were searched against the human SwissProt FASTA database (version May 2019, RRID:SCR_021164), and a common contaminants database downloaded from the Global Proteome Machine in 2019. For the normalization we used Scaled relative abundance (%) – First, the total signal is summed for each TMT channel across all proteins. Normalization factors (typically 1–1.5) are then calculated by scaling each channel's total signal to match that of the highest-signal channel. Each protein's raw summed signal-to-noise value is multiplied by its channel's normalization factor to correct for loading differences. For each protein, the normalized signal is then summed across all TMT channels, and the signal in each individual channel is expressed as a percentage of that protein's total summed signal — giving the scaled relative abundance per channel. This is a default method in Proteome Discoverer.  For the multiplicity procedure we used a multiple ANOVA with permutation-based FDR (0.05) in Perseus, v2.0.3.0 (Max Planck Institute). Batch effects were adjusted using the LIMMA R package built into Perseus, see Supplementary Table 4 for the batch and sample information and imputation using the QRILC R package for Perseus [1]. In total 1209 proteins were identified, where 649 proteins were used for statistical testing after removal of valid values and imputation.

Within patient correlation analysis revealed Pearson correlation coefficients were high across the dataset, with most sample pairs showing correlations closer to 1, indicating a strong overall similarity in protein abundance profiles between samples. Hierarchical clustering of the correlation matrix showed that most samples grouped closely together, indicating overall consistency of the TMT-based quantitative proteomic data. Overall, the correlation analysis indicated good reproducibility across the sample set. There were no missing data.

**Preparation of patient plasma for LC-MS/MS**

Plasma was harvested by centrifugation (2000g at 4°C) from patient blood collected on days 1, 4, 7, 11, 15, and 22 during the first cycle of treatment, Supplementary Table 4. The plasma was then centrifuged at 2000×g for 5 min to remove cell debris, followed by sterile filtration (pore size: 0.22 μm, Millipore). The plasma protein concentration was measured by using the Qubit Protein Assay.

*Trypsin digestion of conditioned medium*

The plasma samples containing 20 μg of protein was buffered with 0.1M Tris pH 7.6, reduced with 20 mM DTT (7min at 95˚C), alkylated with 50 mM iodoacetamide (1h at RT, (dark), and digested overnight at 37°C with 1:50 enzyme: substrate ratio of sequencing grade trypsin (Promega, Madison, WI). Following digestion, samples were acidified with formic acid and desalted using HLB Oasis SPE cartridges (Waters, Milford, MA). Samples were eluted with 80% acetonitrile in 0.1% formic acid, followed by 100% methanol, and concentrated (speedvac). Peptides were stored at -80°C until use.

*TMT labeling of digested and desalted samples*

Samples were resuspended in 100mM HEPES pH 8.5, vortexed and sonicated and aliquoted for labelling using 11 TMT-labels out of 16-plex, resuspended in anhydrous acetonitrile. For 5 μg of peptide in HEPES, TMT-labels were added in a 5:1 ratio (i.e. 25μg), added twice for a total of 50 μg with a 30-minute incubation period. After the second incubation, reaction was stopped by adding Hydroxylamine to a final concentration of 0.4%. Labelled samples were combined by experiment, then desalted using Oasis HLB 10 mg SPE elution plates. After drying, samples were resuspended in a solution of 3%ACN / 10mM Ammonium formate and subjected to offline high-pH reverse fractionation using the Agilent 1260 Infinity HPLC. After concentration, samples were then resuspended in 2%ACN/0.5% FA for LC-MS analysis.

*Electrospray ionization liquid chromatography mass spectrometry (120 minutes)*

Approximately 0.5μg protein tryptic peptides dissolved in 2% acetonitrile (ACN), 0.5% formic acid (FA), were injected into an Ultimate 3000 RSLC nano system (Thermo Scientific, Sunnyvale, California, USA) connected online to a Thermo Orbitrap Exploris 480 mass spectrometer (Thermo Scientific, Bremen, Germany) equipped with EASY-spray nano-electrospray ion source (Thermo Scientific) with FAIMS Pro enabled. The sample was loaded and desalted on a pre-column (Acclaim PepMap 100, 2cm x 75µm ID nanoViper column, packed with 3µm C18 beads) at a flow rate of 5µl/min for 5 min with 0.1% TFA. Peptides were separated during a biphasic ACN gradient from two nanoflow UPLC pumps (flow rate of 250 nl/min) on a 25 cm analytical column (PepMap ES902, 50cm x 75 µm ID EASY-spray column, packed with 2µm C18 beads). Solvent A and B were 0.1% FA (*v/v*) in water and 100% ACN, respectively. The gradient composition was 5% B during trapping (5min) followed by 5-6% B over 0.5min, 6–22% B for the next 69.5 min, 22-32% B over 15 min, and 32–80% B over 2min. Elution of very hydrophobic peptides and conditioning of the column were performed during 10 minutes isocratic elution with 80%B and 15 minutes isocratic conditioning with 5% B. During this process the column valve was changed to inject position (1_2) at 5 min, back to load (6_1) at 105 min.

The eluting peptides from the LC-column were ionized in the electrospray and analyzed by the Thermo Orbitrap Exploris 480 with FAIMS Pro enabled. The FAIMS was operated with 2CVs (CV-45 and CV-60) with cycle times capped to 1.5sec. The mass spectrometer was operated in the DDA-mode (data-dependent-acquisition) to automatically switch between full scan MS and MS/MS acquisition. Instrument control was through Orbitrap Exploris 480 Tune 2.8 and Xcalibur 3.1 Survey full scan MS spectra (from m/z 375-1500) were acquired in the Orbitrap with resolution R = 60000 at m/z 200, automatic gain control (AGC) target of 3e6 and a maximum injection time (IT) set to Auto. The most intense eluting peptides above an intensity threshold of 20 000 counts, and charge states 2 to 6, were sequentially isolated to a target value (AGC) of 2e5 and a maximum IT of 120ms in the C-trap, and isolation width maintained at 0.7 m/z (no offset), before fragmentation in the HCD (Higher-Energy Collision Dissociation) cell. Fragmentation was performed with a normalized collision energy (NCE) of 32 %, and fragments were detected in the Orbitrap at a resolution of 45 000 at m/z 200, with first mass fixed at m/z 110. One MS/MS spectrum of a precursor mass was allowed before dynamic exclusion for 30s with “exclude isotopes” on. Lock-mass internal calibration was not enabled. The spray and ion-source parameters were as follows. Ion spray voltage = 2000V, no sheath and auxiliary gas flow, and capillary temperature = 275 °C. FAIMS carrier gas flow was set to 4.6 L/min.

*Data processing and bioinformatic analysis*

MS raw files were analyzed by the Proteome Discoverer (Version 2.8), and peak lists were searched with Sequest HT against the human SwissProt FASTA database (version May 2019, RRID:SCR_021164), and a common contaminants database downloaded from the Global Proteome Machine in 2019. As variable modification, methionine oxidation was used, and as fixed modification cysteine carbamidomethylation was used. TMT-pro was added as peptide N-terminal modification and lysine modification. False discovery rate was set to 0.01 for proteins and peptides (minimum length of six amino acids) and was determined by the Percolator node Trypsin was set as digestion protease, and a maximum of two missed cleavages were allowed in the database search. Peptide identification was performed with an allowed MS mass deviation tolerance of 20 ppm, and MS/MS fragment ions could deviate by up to 0.02 Da.

**Statistical analysis**

For LC-MS/MS data, hierarchical clustering was performed were performed in MaxQuant Perseus v2.0.3.0 [5] to identify significant Differentially Expressed Proteins (DEPs). Perseus was used using default settings, Euclidian and average linkage. An unsupervised Euclidian hierarchical clustering was performed on the whole dataset. For the multiple samples test an ANOVA with permutation-based FDR (0.05) was performed in Perseus. For a Tukey’s post hoc test we applied the SPSS (version 25, RRID:SCR_016479). Pathway enrichment and functional annotation analyses of these DEPs were conducted using ClueGO v2.5.7, a plug-in tool within Cytoscape (v3.8.2, RRID:SCR_003032) [6], to explore associated biological processes and pathways. This analysis enabled the identification and visualization of functionally grouped significant Gene Ontology (GO), GO (Biological Process) terms, KEGG (Kyoto Encyclopedia of Genes and Genomes), and Reactome pathways. ClueGO analysis was carried out using default parameters. P-values were adjusted for multiple testing using the Bonferroni step-down method and GO terms or pathways with an adjusted p-value (False Discovery Rate, FDR < 0.05) were considered significant. A heatmap generated using SRplot (RRID:SCR_025904) [7] illustrates the expression patterns of the identified DEPs across patient groups during different treatment time points. Additionally, linear mixed effects models were performed for each protein. Responders and non-responders were compared in one model, while differences between treatment timepoint were set up in a separate model. For the treatment timepoint model, Sidak adjustment of p-values was performed to account for multiple comparisons. For the linear mixed models, patient was entered as a random factor (i.e. random intercept model), while the indicator for responders versus non-responders and the categorical variable for treatment timepoint was entered as fixed effects accordingly. As an addendum we calculated Sidak adjustment for the overall p-values, adjusting these for the number of proteins analyzed. Ordinal linear models are also provided so that deviations between the ordinary linear models and the mixed linear models can be examined. Effect estimates and uncertainties are provided in Supplementary Table 3.

**Supplementary Tables (separate files)**

Supplementary Table 1. Individual adverse events for each patient

Supplementary Table 2. Number of adverse events and grading of first *n*=15 BORTEM-17 patients

Supplementary table 3. LC-MS/MS data statistics summary (ANOVA and Linear mix effects model)

Supplementary Table 4. Patient sampling, sample availability, and TMT batch design for the proteomic analysis.

Supplementary Table 5. Summary statistics (Sample correlation analysis)

**References**

1.	Rudolph, J.D. and J. Cox, *A Network Module for the Perseus Software for Computational Proteomics Facilitates Proteome Interaction Graph Analysis.* J Proteome Res, 2019. **18**(5): p. 2052-2064.

2.	Hegi, M.E., et al., *No benefit from TMZ treatment in glioblastoma with truly unmethylated MGMT promoter: Reanalysis of the CE.6 and the pooled Nordic/NOA-08 trials in elderly glioblastoma patients.* Neuro Oncol, 2024. **26**(10): p. 1867-1875.

3.	Louis, D.N., et al., *The 2021 WHO Classification of Tumors of the Central Nervous System: a summary.* Neuro Oncol, 2021. **23**(8): p. 1231-1251.

4.	Wen, P.Y., et al., *RANO 2.0: Update to the Response Assessment in Neuro-Oncology Criteria for High- and Low-Grade Gliomas in Adults.* J Clin Oncol, 2023. **41**(33): p. 5187-5199.

5.	Tyanova, S., et al., *The Perseus computational platform for comprehensive analysis of (prote)omics data.* Nat Methods, 2016. **13**(9): p. 731-40.

6.	Gustavsen, J.A., et al., *RCy3: Network biology using Cytoscape from within R.* F1000Res, 2019. **8**: p. 1774.

7.	Tang, D., et al., *SRplot: A free online platform for data visualization and graphing.* PLoS One, 2023. **18**(11): p. e0294236.
