# Supplementary material for: Contrasting molecular and pharmacodynamic signatures of response and resistance to sequential bortezomib and temozolomide in recurrent IDH-wildtype glioblastoma and grade 4 IDH-mutant astrocytoma: an exploratory interim analysis of NCT03643549 phase IB/II trial
Source: Biomark Res. 2026 Oct 27;14:148. doi: 10.1186/s40364-026-01023-w (PMC13715206; doi:10.1186/s40364-026-01023-w)
Supplement: Supplementary file 3 [file 40364_2026_1023_MOESM5_ESM.docx]

**Supplementary Table 2: Number of adverse events and grading of first *n*=15 BORTEM-17 patients**

| Category | Grades | | | | Total |
| --- | --- | --- | --- | --- | --- |
| Toxicity | **1** | **2** | **3** | **4** |  |
| Blood system disorders | | | | | |
| Platelet count decreased | 4 | 1 | 2 | 2 | 9 |
| Suspected febrile neutropenia | 0 | 0 | 1 | 0 | 1 |
| Anemia | 0 | 1 | 0 | 0 | 1 |
| Gastrointestinal disorders | | | | | |
| Diarrhea/Constipation/abdominal pain | 3 | 1 | 0 | 0 | 4 |
| Dyspepsia | 1 | 0 | 0 | 0 | 1 |
| Nausea | 1 | 1 | 0 | 0 | 2 |
| Vomiting | 1 | 0 | 0 | 0 | 1 |
| Anal hemorrhage | 1 | 0 | 0 | 0 | 1 |
| Gait Disturbance | 1 | 0 | 0 | 0 | 1 |
| Skin and subcutaneous tissue disorders | | | | | |
| Pruritus | 0 | 1 | 0 | 0 | 1 |
| Urticaria | 0 | 1 | 0 | 0 | 1 |
| Nervous system disorders and eye | | | | | |
| Paresthesia | 2 | 1 | 0 | 0 | 3 |
| Depression | 1 | 0 | 0 | 0 | 1 |
| Blurred vision | 1 | 0 | 0 | 0 | 1 |
| Dysarthria | 1 | 0 | 0 | 0 | 1 |
| Headache | 0 | 2 | 0 | 0 | 2 |
| Intracranial hemorrhage | 1 | 0 | 0 | 0 | 1 |
| Psychiatric disorders | | | | | |
| Anxiety | 1 | 1 | 0 | 0 | 2 |
| Insomnia | 0 | 1 | 0 | 0 | 1 |
| Delirium | 0 | 0 | 1 | 0 | 1 |
| Suicidal ideation/ attempt | 1 | 0 | 1 | 0 | 2 |
| Epileptic seizure | 0 | 0 | 3 | 0 | 3 |
| General disorders | | | | | |
| Fatigue | 0 | 5 | 0 | 0 | 5 |
| Fall | 3 | 0 | 1 | 0 | 4 |
| Muscle weakness in the lower limbs | 1 | 0 | 1 | 0 | 2 |
| Hypertension | 1 | 0 | 0 | 0 | 1 |
| Urinary incontinence | 1 | 0 | 0 | 0 | 1 |
| Infections | | | | | |
| Upper respiratory infection | 1 | 0 | 0 | 0 | 1 |
| Gastrointestinal infection | 1 | 0 | 0 | 0 | 1 |
| Urinary tract infection | 1 | 0 | 0 | 0 | 1 |
| Metabolism and Nutrition Disorders | | | | | |
| Anorexia | 0 | 1 | 0 | 0 | 1 |
| Hyperglycemia | 0 | 0 | 1 | 0 | 1 |
| Hypophosphatemia | 0 | 1 | 0 | 0 | 1 |
| Hypokalemia | 1 | 0 | 0 | 0 | 1 |
| Total | 30 | 18 | 11 | 2 | 61 |

Adverse events (AEs) were categorized according to MedDRA system organ classes and graded for severity using the National Cancer Institute Common Terminology Criteria for Adverse Events (CTCAE), version 4.03 in n=15 patients. Values represent the number of events by grade (1–4)
