# Supplementary material for: Contrasting molecular and pharmacodynamic signatures of response and resistance to sequential bortezomib and temozolomide in recurrent IDH-wildtype glioblastoma and grade 4 IDH-mutant astrocytoma: an exploratory interim analysis of NCT03643549 phase IB/II trial
Source: Biomark Res. 2026 Oct 27;14:148. doi: 10.1186/s40364-026-01023-w (PMC13715206; doi:10.1186/s40364-026-01023-w)
Supplement: Supplementary file 4 [file 40364_2026_1023_MOESM7_ESM.docx]

**Supplementary Table 4. Patient sampling, sample availability, and TMT batch design for the proteomic analysis.**

S.Table 4a. Patients and proteomic sampling schedule

| Patients | # of patients (n) | Sample available for measurements (timepoints) | Samples per patients |
| --- | --- | --- | --- |
| P01/01- P01/10 | 10 | Baseline, D1 4h, D1 24h, D4+D7, D7 4h, D11, D15, D22 | 8 |
| P01/11- P01/15 | 5 | Baseline, D1 4h, D4, D22 | 4 |
| Total | 15 |  | 100 Samples |

S.Table 4b. Availability of plasma samples by patients and time points

| Patients | Base | D1 4h | D1 24h | D4 | D4+D7 | D7 4h | D11 | D15 | D22 |
| --- | --- | --- | --- | --- | --- | --- | --- | --- | --- |
| P01/01-P01/10 | Yes | Yes | Yes | NA | Yes | Yes | Yes | Yes | Yes |
| P01/11-P01/15 | Yes | Yes | NA | Yes | NA | NA | NA | NA | Yes |
| n | 15 | 15 | 10 | 5 | 10 | 10 | 10 | 10 | 15 |

Treatment category:

Base = Baseline (Before Treatment)

D1 4h and D1 24h = BTZ only

D4+D7 and D4 = TMZ only

D7 4h = BTZ+TMZ

D11, D15 and D22 = Recovery

S.Table 4c. Number of proteomic samples analyzed at each time point

| Time-point label | Patients | # of samples analyzed |
| --- | --- | --- |
| Baseline | P01/01- P01/15 | 15 |
| D1 4h | P01/01- P01/15 | 15 |
| D1 24h | P01/01- P01/10 | 10 |
| D4 | P01/11- P01/15 | 5 |
| D4+D7 | P01/01- P01/10 | 10 |
| D7 4h | P01/01- P01/10 | 10 |
| D11 | P01/01- P01/10 | 10 |
| D15 | P01/01- P01/10 | 10 |
| D22 | P01/01- P01/15 | 15 |

S.Table 4d. TMT labels allocation for samples across the 12 batches (EX1–EX12)

| **TMT Labels** | **EX1** | **EX2** | **EX3** | **EX4** | **EX5** | **EX6** | **EX7** | **EX8** | **EX9** | **EX10** | **EX11** | **EX12** |
| --- | --- | --- | --- | --- | --- | --- | --- | --- | --- | --- | --- | --- |
| **126** | P01/01 Base | P01/02 Base | P01/03 Base | P01/04 Base | P01/05 Base | P01/06 Base | P01/07 Base | P01/08 Base | P01/09 Base | P01/10 Base | P01/11 Base | P01/12 Base |
| **127N** | P01/01 D1 4h | P01/02 D1 4h | P01/03 D1 4h | P01/04 D1 4h | P01/05 D1 4h | P01/06 D1 4h | P01/07 D1 4h | P01/08 D1 4h | P01/09 D1 4h | P01/10 D1 4h | P01/11 D1 4h | P01/12 D1 4h |
| **127C** | P01/01 D1 24h | P01/02 D1 24h | P01/03 D1 24h | P01/04 D1 24h | P01/05 D1 24h | P01/06 D1 24h | P01/07 D1 24h | P01/08 D1 24h | P01/09 D1 24h | P01/10 D1 24h | P01/11 D4 | P01/12 D4 |
| **128N** | P01/01 D4+D7 | P01/02 D4+D7 | P01/03 D4+D7 | P01/04 D4+D7 | P01/05 D4+D7 | P01/06 D4+D7 | P01/07 D4+D7 | P01/08 D4+D7 | P01/09 D4+D7 | P01/10 D4+D7 | P01/11 D22 | P01/12 D22 |
| **128C** | P01/01 D7 4h | P01/02 D7 4h | P01/03 D7 4h | P01/04 D7 4h | P01/05 D7 4h | P01/06 D7 4h | P01/07 D7 4h | P01/08 D7 4h | P01/09 D7 4h | P01/10 D7 4h | P01/13 Base | P01/14 Base |
| **129N** | P01/01 D11 | P01/02 D11 | P01/03 D11 | P01/04 D11 | P01/05 D11 | P01/06 D11 | P01/07 D11 | P01/08 D11 | P01/09 D11 | P01/10 D11 | P01/13 D1 4h | P01/14 D1 4h |
| **129C** | P01/01 D15 | P01/02 D15 | P01/03 D15 | P01/04 D15 | P01/05 D15 | P01/06 D15 | P01/07 D15 | P01/08 D15 | P01/09 D15 | P01/10 D15 | P01/13 D4 | P01/14 D4 |
| **130N** | P01/01 D22 | P01/02 D22 | P01/03 D22 | P01/04 D22 | P01/05 D22 | P01/06 D22 | P01/07 D22 | P01/08 D22 | P01/09 D22 | P01/10 D22 | P01/13 D22 | P01/14 D22 |
| **131C** |  |  |  |  | P01/15 base | P01/15 D1 4h | P01/15 D4 | P01/15 D22 | REF | REF | REF | REF |
| **132N** | REF | REF | REF | REF | REF | REF | REF | REF | REF | REF | REF | REF |
| **132C** | REF | REF | REF | REF | REF | REF | REF | REF | REF | REF | REF | REF |

S.Table 4e. Distribution of patient samples for 12 TMT proteomic batches

| **Batch** | **Samples** |
| --- | --- |
| 1 | P01/01 (8 samples) |
| 2 | P01/02 (8 samples) |
| 3 | P01/03 (8 samples) |
| 4 | P01/04 (8 samples) |
| 5 | P01/05 (8 samples) + P01/15 baseline |
| 6 | P01/06 (8 samples) + P01/15 D1 4h |
| 7 | P01/07 (8 samples) + P01/15 D4 |
| 8 | P01/08 (8 samples) + P01/15 D22 |
| 9 | P01/09 (8 samples) |
| 10 | P01/10 (8 samples) |
| 11 | P01/11 (4 samples) + P01/13, (4 samples) |
| 12 | P01/12 (4 samples) + P01/14, (4 samples) |
